# A Review of Current Trends on Polyvinyl Alcohol (PVA)-Based Solid Polymer Electrolytes

**DOI:** 10.3390/molecules28041781

**Published:** 2023-02-13

**Authors:** John Ojur Dennis, M. F. Shukur, Osamah A. Aldaghri, Khalid Hassan Ibnaouf, Abdullahi Abbas Adam, Fahad Usman, Yarima Mudassir Hassan, A. Alsadig, Wilson L. Danbature, Bashir Abubakar Abdulkadir

**Affiliations:** 1 Department of Fundamental and Applied Sciences, Universiti Teknologi PETRONAS, Seri Iskandar 32610, Malaysia; 2 Department of Physics, College of Science, Imam Mohammad Ibn Saud Islamic University (IMSIU), Riyadh 13318, Saudi Arabia; 3 Department of Physics, Al-Qalam University, Katsina PMB 2137, Nigeria; 4 CNR Nanotec, University Campus Ecotekne, 73100 Lecce, Italy; 5 Department of Chemistry, Gombe State University, Gombe PMB 127, Nigeria

**Keywords:** polyvinyl alcohol, solid polymer electrolytes, supercapacitors, ionic conductivity

## Abstract

Presently, the rising concerns about the fossil fuel crisis and ecological deterioration have greatly affected the world economy and hence have attracted attention to the utilization of renewable energies. Among the renewable energy being developed, supercapacitors hold great promise in broad applications such as electric vehicles. Presently, the main challenge facing supercapacitors is the amount of energy stored. This, however, does not satisfy the increasing demand for higher energy storage devices, and therefore, intensive research is being undertaken to overcome the challenges of low energy density. The purpose of this review is to report on solid polymer electrolytes (SPEs) based on polyvinyl alcohol (PVA). The review discussed the PVA as a host polymer in SPEs followed by a discussion on the influence of conducting salts. The formation of SPEs as well as the ion transport mechanism in PVA SPEs were discussed. The application and development of PVA-based polymer electrolytes on supercapacitors and other energy storage devices were elucidated. The fundamentals of electrochemical characterization for analyzing the mechanism of supercapacitor applications, such as EIS, LSV and dielectric constant, are highlighted. Similarly, thermodynamic transport models of ions and their mechanism about temperature based on Arrhenius and Vogel–Tammann–Fulcher (VTF) are analyzed. Methods for enhancing the electrochemical performance of PVA-based SPEs were reported. Likely challenges facing the current electrolytes are well discussed. Finally, research directions to overcome the present challenges in producing SPEs are proposed. Therefore, this review is expected to be source material for other researchers concerned with the development of PVA-based SPE material.

## 1. Introduction

Presently, the rising and increasing concerns about the fossil fuel crisis and ecological deterioration have greatly affected the world economy and hence have attracted significant attention to the utilization of renewable energies such as wind, solar and tidal [1]. However, the production of these energies is occasional and comes with lots of drawbacks such as low power output [2]. Similarly, the demand for energy is not coping with the supply of the same. Therefore, energy storage has become one of the critical subjects which lead to better utilization of sustainable energies in the present and the future for environmentally friendly high-power energy resources [3,4]. Among several energy storage devices and technologies, lithium-ion batteries (LIBs) and supercapacitors hold great promise in broad applications such as electrical vehicles, smart grids and portable electronics. LIBs, as a kind of secondary batteries, have been commonly used in people’s daily lives since Sony Company first commercialized them in 1991. LIBs have a high energy density, around 150~200 Wh/kg, which enables them to store electrical energy in lightweight devices [5]. Hence, LIBs are widely used not only in portable electronics but also in aircraft and automotive vehicles, which are commonly powered by fossil fuels. In addition, compared to conventional secondary batteries, which suffer from the memory effects, LIBs can be charged at any time even after being only partially discharged [6]. However, there are still some technical challenges posed by LIB batteries, such as the inability to respond to sudden high-power demand due to their low power density [1,7]. The power density of energy storage systems plays an important role in starting up diesel locomotives and accelerating a hybrid vehicle. Furthermore, the electrolyte in LIBs containing flammable organic solvents such as dimethyl carbonate (DMC), diethyl carbonate (DC) and ethylene carbonate (EC) are reported to be highly hazardous. Consequently, explosions have been experienced in some devices utilizing LIBs resulting in serious safety issues and even death [8].

Another serious concern regarding the use of LIBs is related to battery life. Based on manufacturers’ information, the performance of LIBs drops to about 80% after 500 cycles, signifying that the batteries of phones that are used daily can barely last for more than three years [8]. Furthermore, for rechargeable batteries, a short recharge time is important, especially for electric cars. For example, it was reported that a Tesla electric car battery can charge at the rate of 92 km of range per hour with a wall connector and dual chargers, which make it less suitable for long-distance travelling than traditional cars [8,9]. Yet, when a single charger and mobile connector are utilized, the charge rate is even lower. Therefore, extensive adoption of environmentally friendly electrical vehicles is still hindered by the long charge time of LIBs. LIBs have energy densities of at least two orders of magnitude lower as compared to fossil fuels [10]. Hence, to replace fossil fuels with LIBs, a large quantity of these batteries is required, which leads to an increase in mass production [11].

**Figure 1:**
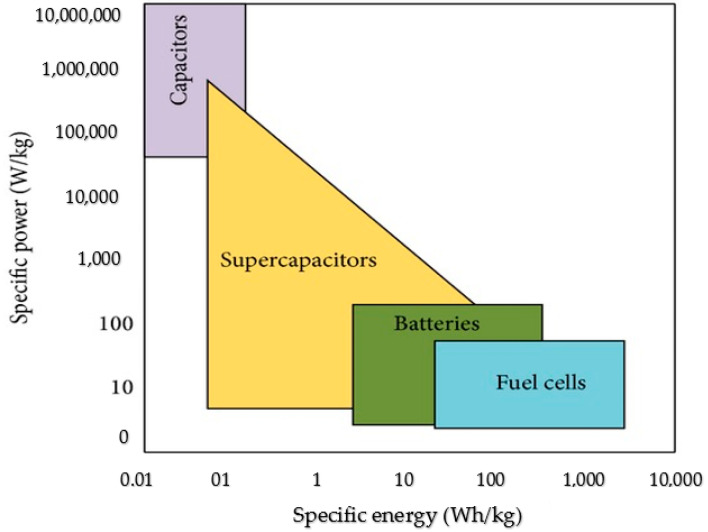
Ragone plot comparing main energy storage devices of fuel cells, batteries, supercapacitors and capacitors [2].

Thus, supercapacitors, also known as ultracapacitors, are seen to be a promising substitute or complementary technology for future energy storage devices because of their long cycle life, short charging time and high performance [1]. Figure 1 shows the Ragone plot for the comparison of fuel cells, batteries, supercapacitors and capacitors [2]. Supercapacitors occupy an extremely imperative position in terms of specific energy and specific power in the plot. They have better performance than LIBs and conventional capacitors in terms of specific power and specific energy, respectively, and therefore, they are the best option for the rising demands of the next-generation energy storage systems [3].

Presently, the main challenge facing electrochemical supercapacitors when compared with fuel cells and batteries is their amount of energy stored. It is reported that the energy stored by electrochemical supercapacitors is generally between 20 Wh kg^−1^ and 50 Wh kg^−1^ [4]. This, however, does not satisfy the increasing demand for higher energy storage devices, and therefore, intensive research is being undertaken to overcome the challenges of low energy density in supercapacitors.

**Figure 2:**
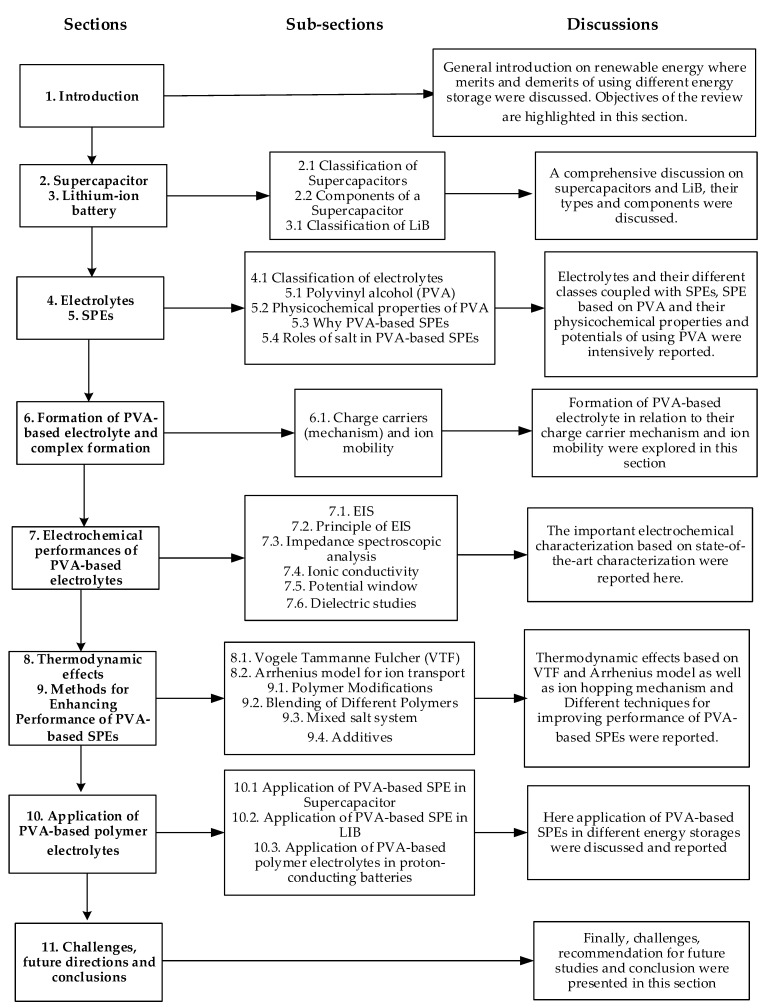
Schematic summary of the article.

The purpose of this review, after the brief introduction on electrochemical supercapacitors, is to give a review on solid polymer electrolytes (SPEs) based on polyvinyl alcohol (PVA) for electrochemical supercapacitors. It begins with a description of PVA as a host polymer in SPEs followed by a discussion on the influence of conducting salts in PVA. The formation of SPEs as well as the ion transport mechanism (charge carrier) in PVA SPEs are extensively discussed. Application and development of PVA-based polymer electrolytes on supercapacitors and other energy storage devices such as LIBs and proton-conducting batteries were elucidated. The fundamentals of electrochemical characterization for analyzing the mechanism of supercapacitor applications, such as electrochemical impedance spectroscopy (EIS), ionic conductivity, linear sweep voltammetry (LSV) and dielectric constant are highlighted. Similarly, thermodynamic transport models of ions and their mechanism in relation to temperature based on Arrhenius and Vogel–Tammann–Fulcher (VTF) are clearly analyzed. Methods for enhancing the electrochemical performance of PVA-based SPEs such as polymer modifications, blending of different polymers; mixed salt systems and the use of additives (fillers/plasticizers) are reported. Likely challenges facing the current electrolytes are well discussed. Finally, research directions to overcome the present challenges in producing highly conducting SPEs are proposed in order to improve the power and energy density of solid-state electrochemical supercapacitors. In this review, we focus mainly on SPEs developed and synthesized based on PVA due to its numerous advantages, such as availability, environment friendliness, less cost, good compatibility and miscibility with salts and other components. Figure 2 shows a schematic summary of the article.

## 2. Supercapacitor

Supercapacitors (SCs), also named electrochemical capacitors or ultracapacitors, are electrical components or energy storage devices that are able to store and accommodate definite amounts of energy. The development of supercapacitors dates back to the 1950s of the 20th century. The experiments started between the 1950s and 1970s and were conducted by US companies General Electric (GE) and Standard Oil of Ohio (SOHIO), where the first electrochemical supercapacitors reached the capacity of around 1 F. The first supercapacitor was named “Gold Cap” and it was commercialized in 1982 by Panasonic Company. The Pinnacle Research Institute (PRI) developed the first electric double-layer capacitor (EDLC) for military purposes, in the year 1982. SCs are energy storage devices that are used to bridge the gap between batteries and conventional capacitors which can store more energy than capacitors and supply it at a higher energy density than batteries. These features, coupled with good cyclability and long-term stability, make SCs attractive devices for energy storage. SCs are already present in many applications, either in combination with other energy storage devices (mainly batteries) or as autonomous energy sources [12,13,14,15,16].

**Figure 3:**
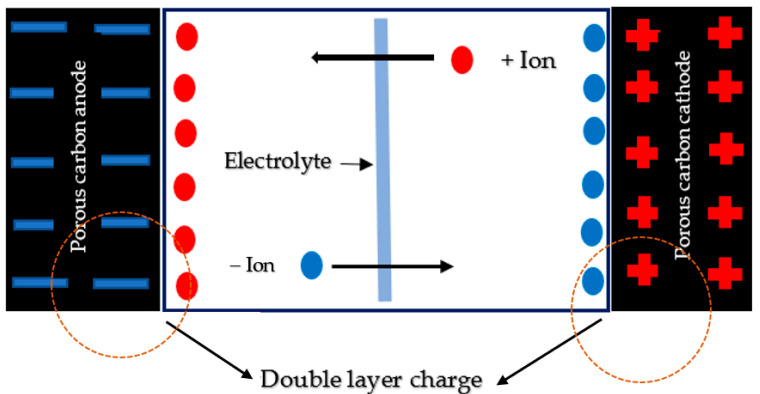
Schematic illustration of a typical charged EDLC [20,21].

SCs are used in applications, where storing or releasing a large amount of energy is required. Nowadays, the SCs are used primarily in Hybrid Electric Vehicles (HEV), Electric Vehicles (EV) and Fuel Cell Vehicles (FCV) such as passenger cars, trains, and trolleybuses. Other areas of SCs use are electronic devices such as Uninterruptible Power Supplies (UPS), flexible devices such as handphones, and volatile memory backups in PCs. Similarly, supercapacitors can be used to increase the efficiency of hybrid electric vehicles in several ways [17,18]. SCs store energy by the accumulated oppositely charged ions from the electrolyte on the high surface area of the two electrodes when a voltage is applied. The positively charged ions move and align towards the negative electrode, while the negatively charged ions align towards the positive electrode, thereby producing a double layer charge, as shown in Figure 3. The applied voltage is always less than the required voltage capable of causing a chemical reaction. Hence, no chemical changes occur in the system. Due to the complete physical mechanism, the SCs are very stable and maintain a very high cycle life of up to 10^6^ [19,20].

### 2.1. Classification of Supercapacitors

**Figure 4:**
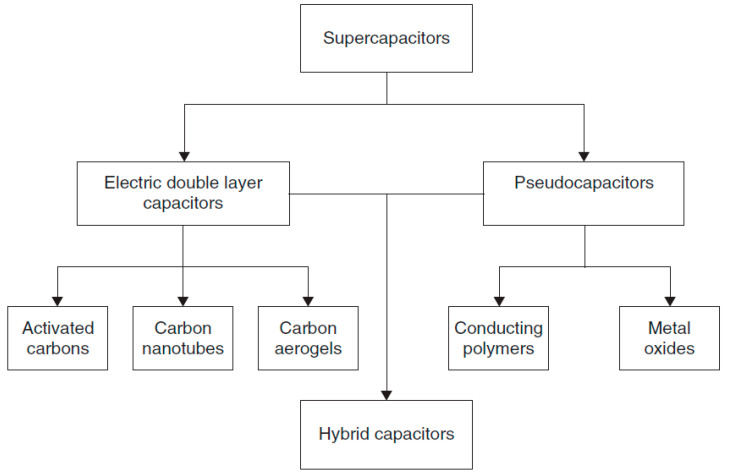
Block diagram for types of supercapacitors.

Generally, SCs can be classified into three major groups according to the transport mechanism, electrochemical double-layer capacitors (EDLCs), pseudocapacitors and hybrid capacitors, as shown in Figure 4. The capacitance in EDLC, which usually uses activated carbon as the electrode, comes from the electrostatic charge that is stored at the surface of the system [5,6]. Pseudocapacitors’ charging mechanism can be categorized according to reversible and fast Faradaic redox reactions through electrode materials, for instance, oxides of transition metals and electrically conducting polymers [2]. It was reported that the energy density of pseudocapacitance is found to be higher than that of EDLCs. Nevertheless, phase changes arising at the electrode through the charge and discharge process owing to the Faradaic reaction are a serious setback to the power density [2,5]. The third category of the supercapacitor is the hybrid supercapacitor, where the synergetic effect and function of both EDLC and pseudocapacitance can be attained simultaneously.

**Figure 5:**
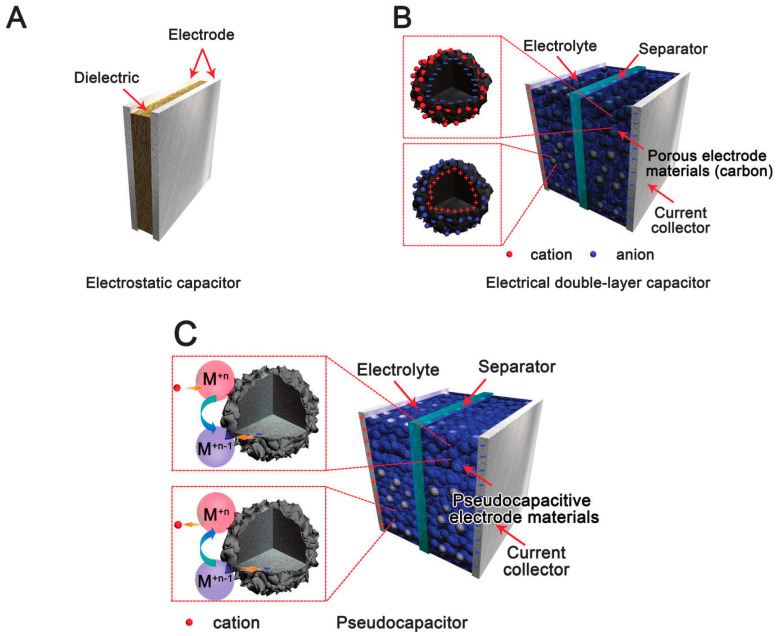
Schematic diagram of (**A**) an electrostatic capacitor, (**B**) an electric double-layer capacitor and (**C**) a pseudocapacitor [17].

Fundamentally, a supercapacitor is a special type of capacitor, which is different from the classical electrostatic capacitors, as shown in Figure 5. It can be distinguished in several ways, such as the charge storage mechanism, the electrolyte, the electrode material and the cell structure [17].

### 2.2. Components of a Supercapacitor

**Figure 6:**
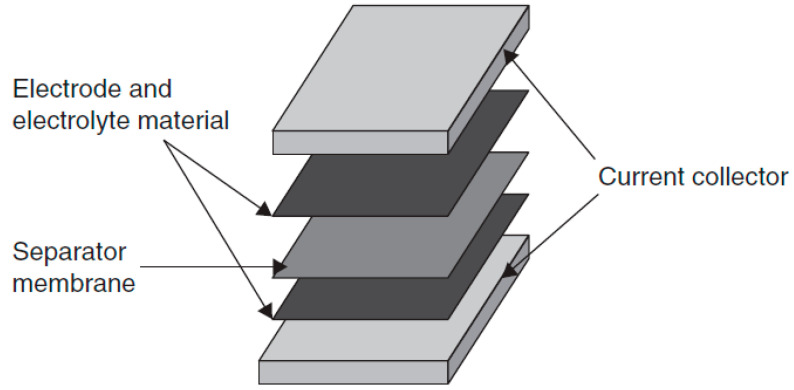
The three major constituents of a supercapacitor.

A supercapacitor consists of three main components, namely, electrodes, separators, and electrolytes, as illustrated in Figure 6. The electrode is an electrical conductor that contacts the electrolyte. Carbon material (mostly porous) is reported to be suitable material for use as a supercapacitor electrode owing to several advantages such as chemical and physical stability [7,8]. Among supercapacitor carbon electrode materials widely explored are activated carbon (AC), carbon nanotubes (CNTs) and graphene [3]. Nowadays, porous carbon is recognized as the most suitable leading electrode material for supercapacitors due to its excellent characteristic including large surface area, high electrical conductivity and good physical and chemical stability [22,23]. Similarly, an ordered pore in the carbon electrode materials can effectively combine the typical roles of most pores/channels, which serve as the ion-buffering reservoirs (macro-pores), fast ion transporting channels (mesopores) and ion-storage sites (micro-pores) [24]. For a perfect carbon electrode, a large surface area can provide enough electrode/electrolyte interfaces for ion or charge storage [25].

SCs based on graphene materials suffer from low specific capacitance and energy density (5–8 Wh kg^−1^), which limits their practical applications [26]. The cost of the materials is another source of concern. Moreover, large-scale production of high-quality graphene is reported to be a huge challenge to researchers, including mechanical exfoliation, epitaxial growth on silicon carbide or metal surface, reduction of graphene oxide (GO) and others [7]. Though chemical reduction of exfoliated GO is commonly regarded as an efficient technique for the low-cost and bulk production of graphene sheets, irreversible aggregation of graphene sheets during reduction is inevitable, and it will cause a possible decrease in the effective surface area and affect their electrochemical properties [7,27]. Therefore, researchers are sparing no efforts to study the electrode materials from AC.

Another important component in a supercapacitor is the separator. A separator is a thin electrically insulating film that is sandwiched between the electrodes. Separators must prevent electrical contact between electrodes but also allow the ions to transfer during the charge and discharge process. Ideal separators for application in supercapacitors will have high electrical resistance, good ionic permeability, and minimum thickness [9]. The most common materials used to construct separators are rubber, plastic, aqua gel, paper, nafion, polypropylene, and polyolefin films [10]. Under certain circumstances, the electrolyte itself acts as the separator membrane as well. The choice of a separator depends on the electrolyte used; glass or ceramic fiber are mostly used for aqueous electrolytes, and polymer/paper separators are for organic electrolytes [9,11].

The last important component of the supercapacitor is electrolytes. Electrolytes have been recognized as one of the most significant components in the performance of SCs and this includes electrical EDLC, pseudocapacitors and hybrid supercapacitors [8]. Electrolytes in SCs have an important role in establishing critical properties in many areas, such as in determining the role of the operating voltage window in electrochemical supercapacitors’ energy density, internal resistance, cycling lifetime, power density, operating temperature range, rate performance, self-discharge and toxicity, which are all important in the practical use of EDLCs [8,28]. The SCs from carbon-based materials are also strongly dependent on the nature of the electrolytes. The ionic conductivity of electrolytes plays a significant role in the internal resistance of SCs [28]. Electrolytes occupy the space in between the electrodes and allow ions to move through them. It is a notably important component in constructing a supercapacitor because the highest operating voltage depends on the breakdown voltage of the electrolyte, which is squarely proportional to both the energy and the power densities of the supercapacitor (Equation (1)) [29]. The electrolyte also affects the equivalent series resistance (ESR), which is a determinant of the power density.

(1) $$P=\frac{1}{4W_{em}R_{cell}}V^{2}$$

where *R_cell_* is the equivalent series resistance (ESR) (Ω).

## 3. Lithium-Ion Battery (LiB)

**Figure 7:**
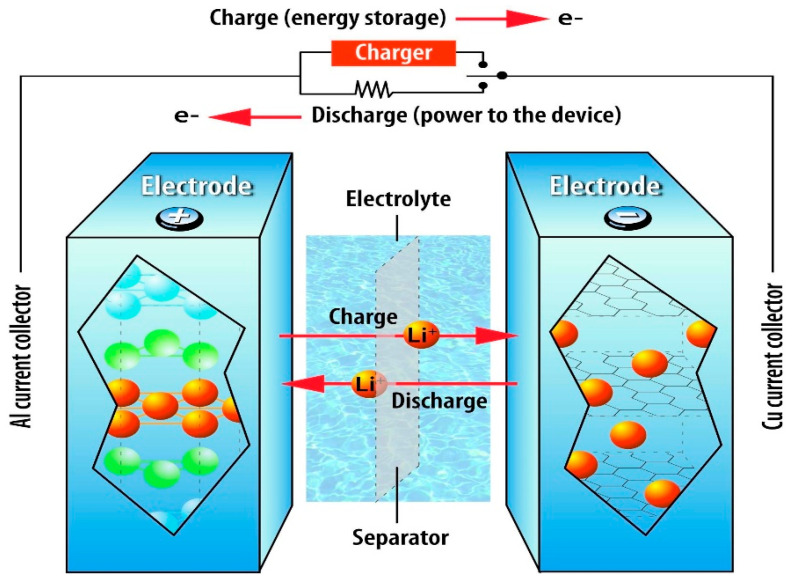
Li-ion battery charge–discharge process [31].

A lithium-ion battery, often known as a Li-ion battery (LiB), is a type of rechargeable battery that stores energy through the reversible reduction of lithium ions. One of the most widely used types of energy storage in the world, lithium-ion batteries accounted for 85.6% of installed energy storage systems in 2015 [30,31]. The batteries have a positive electrode made of lithium metal oxides, which can store lithium ions, and a negative electrode made of carbon. Metal salts, mostly lithium salts that have been dissolved in organic carbonates, serve as the electrolyte. The two-phase transfer of lithium ions is how lithium-ion batteries work. Lithium ions go from the positive electrode to the negative electrode during charging, and the reverse happens during discharging, as shown in Figure 7. Li-ion batteries do not need to be monitored for the temperature to function properly [32]. Li-ion batteries are in high demand because of their availability and dependability and are used in a variety of technologies, including electric vehicles, hybrid electric vehicles, and portable electronics. It is the most common kind of battery used in electric vehicles and portable consumer gadgets. Additionally, grid-scale energy storage as well as military and aerospace applications make major use of it. LiBs outperform other rechargeable battery technologies in terms of energy density, self-discharge, and memory effect [33].

The microelectronics revolution has been facilitated by lithium-ion batteries, which are now the preferred power source for portable electronics. Because they offer higher gravimetric and volumetric energy densities than conventional rechargeable technologies, they have prevailed in the portable electronics market. The use of water-free, nonaqueous electrolytes results in greater operating voltages of 4 V than the use of aqueous electrolytes in other systems, which generally limit the operating voltages to 2 V [33]. This results in a higher energy density. In addition to making inroads into the electric vehicle sector, lithium-ion batteries are also being aggressively pursued in grid energy storage. When using lithium-ion batteries for diverse purposes, there are a number of factors that must be taken into account, including energy, power, charge–discharge rate, cost, cycle life, safety and environmental impact. Cost, cycle life and safety also become crucial factors along with energy density (driving distance between charges) for electric vehicles, even if energy density is the most significant issue for portable devices. However, cost, cycle life and safety take precedence over energy density for grid-energy storage. Despite the cost of such storage systems, the benefits of Li-ion batteries may encourage their adoption in the creation of large-scale storage systems. Moreover, despite being the most expensive battery-type energy storage system, Li-ion batteries have the capacity to store renewable energy due to their low cost per cycle [34].

### Classifications of Lithium-Ion Batteries

**Figure 8:**
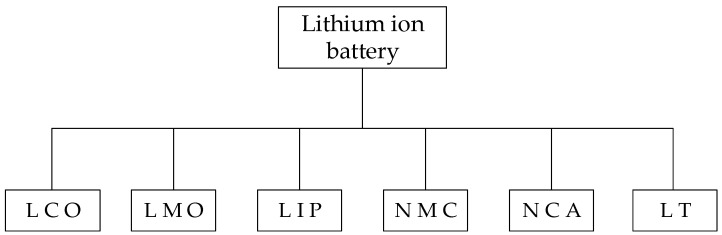
Block diagram for types of Li-ion batteries.

There are various sizes and shapes of lithium-ion batteries, and not all of them are equal. As summarized in Figure 8, six different types of lithium-ion batteries are described here, along with information on their makeup and typical uses. Batteries made of lithium cobalt oxide (LCO), also referred to as lithium cobalt or lithium-ion cobalt batteries, are produced using lithium carbonate and cobalt. Because of their high specific energy, these batteries are employed in electronic cameras, laptops, and cell phones. These types of batteries have a cobalt oxide cathode and a graphite carbon anode. Short battery life and low specific power are two drawbacks of this battery. Despite this, because of their qualities, they are a common option for cell phones and other portable electronic devices. Lithium manganese oxide (LMO) batteries include lithium manganate, lithium-ion manganese, li-manganese and manganese spinel batteries. This type of battery’s technology was first uncovered in the 1980s. The first commercial lithium-ion cells were created by Moli Energy in 1996, utilizing LCO as the cathode material. LCO batteries are safer than other types of LiBs due to their high-temperature stability [32,35]. They can therefore be utilized in power tools, electric motorbikes, and other applications, and are widely found in medical equipment and devices. Electric cars and laptop computers can both be powered by LCOs. In lithium iron phosphate (LIP) batteries, phosphate is employed as the cathode. They are more thermally stable and safe thanks to their low resistance characteristics. Other benefits include longevity and durability; fully charged batteries can be stored without affecting their overall charge life. LIP batteries are typically the most economical choice when the long battery life is taken into account. On the other hand, the lithium-phosphate battery has less energy than conventional lithium batteries, due to its lower voltage. Because of this, these batteries are commonly used in electric motorcycles and other applications that need high levels of safety and a long lifecycle [33].

Nickel and manganese lithium cobalt oxide batteries, also referred to as NMC batteries, are made of a wide range of components that are common to the other batteries. The cathode is made from a combination of nickel, manganese and cobalt. NMC batteries can have a high specific energy density or a high specific power. The typical cathode combination ratios are 60% nickel, 20% manganese and 20% cobalt. As cobalt may be fairly expensive, this means that the cost of the raw material is cheaper than for other lithium-ion battery alternatives. This battery type is frequently utilized in electric vehicles because of its low self-heating rate [35]. Lithium nickel cobalt aluminum (NCA) oxide batteries, are mostly used in electric vehicles. Although NCA batteries are not frequently used in consumer gadgets, the automotive sector has huge potential for them. Although NCA batteries are more expensive and less safe than other forms of lithium-ion batteries, they do provide a high-energy option with a long lifespan. The demand for these batteries may rise as the number of electric vehicles rises, given the extensive use of NCA batteries in electric vehicles. Finally, lithium titanate (LT) is a form of battery that is finding more and more applications. The LT battery’s better nanotechnology facilitates incredibly quick recharging. Electric vehicles, bicycle manufacturers and electric buses use this type of battery. The inability of these batteries to power automobiles as effectively as other types of batteries may result from their lower voltage or energy density. Despite this, there is a benefit in that LT batteries have a higher density than non-lithium-ion batteries. These batteries could be employed in smart grid construction, military and aerospace applications, as well as wind and solar energy storage [35,36].

## 4. Electrolytes

Electrolytes are materials that generally exist as solutions of acids, bases or salts. However, it was reported that some might exist as gas electrolytes under specific conditions such as low pressure or high temperature [11]. Electrolytes can be formed by the dissolution of some biological polymers such as polypeptides and synthetic polymers such as sulfonated polymers [6]. Generally, an ideal electrolyte must possess the following: (1) a wide potential window; (2) a high ionic conductivity; (3) a high chemical and electrochemical stability; (4) high chemical and electrochemical inertness to SCs components (e.g., electrodes, current collectors and packaging); (5) a wide operating temperature range; (6) it must be well-matched with the electrode materials; (7) low volatility and flammability; (8) environmentally friendly; and (9) a low cost.

The electrolyte is an imperative and critical part of SCs and assumes a vital job in moving and adjusting charges among the terminals (electrodes) [2]. Besides the effect of the electrolyte in determining the conductivity and potential window, an electrode–electrolyte interaction during the charge and discharge process assumes a critical effect on the SC’s performance. Compatibility between electrodes and the electrolyte, for example, was reported to play a significant role in the capacitance of the ultracapacitor [37].

### Classification of Electrolytes

**Figure 9:**
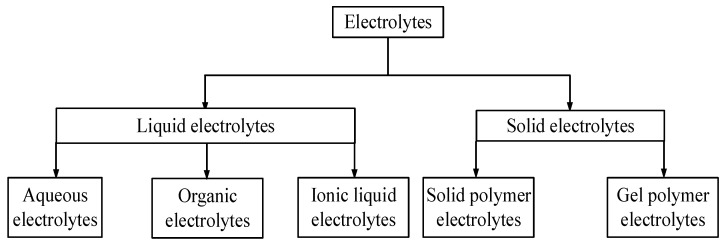
Type of electrolytes used for supercapacitors.

Figure 9 summarized the classification of electrolytes into liquid and solid electrolytes [4,12,38]. Generally, liquid electrolytes are found to have higher conductivity, and the use of liquid electrolytes has significant limitations and drawbacks that include environmental unfriendliness, leakage problems, low cycle life, long charging time, and low potential window [39]. Hence, the improvement of new sorts of supercapacitors, for example, adaptable, solid-state and miniaturized scale supercapacitors, additionally depend profoundly on new electrolytes, for example, solid electrolytes.

## 5. Solid Polymer Electrolytes (SPEs)

The rigorous research effort focused towards SPEs particularly involving polymer electrolytes was mainly driven by their improved safety and applicability in supercapacitors that requires high mechanical flexibility, power density and energy density [40]. One of the key areas of priority in any energy storage device is safety and reusability [41]. The safety issues relating to state-of-the-art supercapacitors are associated with the use of mostly flammable electrolytes. It was found that liquid electrolytes offer high ionic conductivity and good interfacial contact; however, liquid electrolytes were reported to exhibit high reactivity and have serious leakage issues [42,43]. The exothermic reactions occurring at the electrode surface, when using liquid electrolytes, increase the overall temperature of the energy storage very quickly, causing more electrolyte decomposition as well as generating flammable gases that are hazardous to the environment. This is referred to as the notorious thermal runaway [44]. At this stage, the electrolyte is exposed to the air when the device ruptures and the resultant exothermic reaction results in fire, smoke and even explosions, which may lead to death in some cases [45]. In contrast, SPEs are electrochemically stable, which is favorable in decreasing the heat generation of the storage device in case of thermal shock [46]. Similarly, the demands of the consumer for convenient and smart electronics have led to the development of flexible and wearable devices, such as flexible phones, smart watches, electronic clothing, sensitive robotic skins and other mini-wearable devices [47]. SPEs have a tremendous advantage over other technologies since the polymeric backbone of an SPE offers the mechanical flexibility needed for such batteries [48].

Consequently, with growing demand in recent years for portable, wearable and flexible electronic devices, many authors preferred solid electrolytes because of their flexibility and simplicity. These types of electrolytes do not serve as conducting materials only but can serve as a separator to avoid short circuits. With the advances in electrolyte science and technology coupled with serious disadvantages in using liquid electrolytes, various novel solid electrolytes have emerged for supercapacitor application [4,49]. One of these materials, polymer electrolyte, is a material that is ionically conducting, while the electronic conductivity is insignificant.

**Figure 10:**
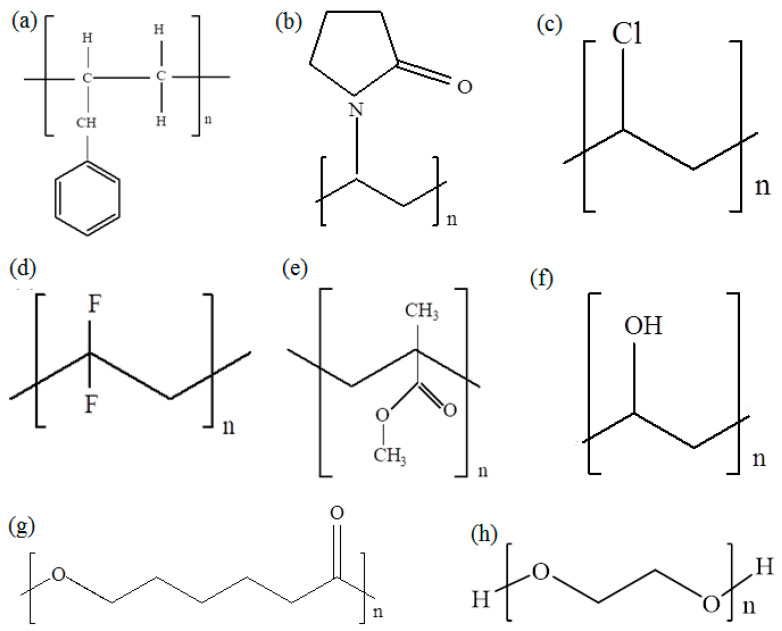
Chemical structures of important polymers mostly applied for solid electrolytes (**a**) PS, (**b**) PVP, (**c**) PVC, (**d**) PVDF, (**e**) PMMA, (**f**) PVA, (**g**) PCL and (**h**) PEO [27].

Intensive studies are using various polymers as host materials in SPEs due to the simplicity of their synthesis, high conductivity, cost efficiency, and lightweight. Some of the polymers that have been mostly used in solid electrolytes are: polystyrene (PS), poly(ethylene oxide) (PEO), Polyvinyl alcohol (PVA), Poly(3-caprolactone) (PCL), poly (methyl methacrylate) (PMMA), Poly(vinyl chloride) (PVC), Poly(vinylpyrrolidone) (PVP), Chitosan (CS) and poly (vinylidene fluoride) (PVDF) [27]. Figure 10 presents the chemical structures of such important polymers.

### 5.1. Polyvinyl Alcohol (PVA)

Poly(vinyl alcohol), often abbreviated as PVA, PVAL or PVOH, is a synthetic polymer that was first prepared by Hermann and Haehnel, in 1924. PVA is a synthetic biopolymer that is produced by polyvinyl acetate hydrolysis. Because of its water solubility, it is used in a wide range of commercial applications such as protective coatings, binding of pigments and the production of other synthetic polymers. The polymer consists of a carbon backbone with OH side chains and has the chemical formula [CH_2_CH(OH)]_n_. The application of biopolymer electrolytes using biodegradable polymers such as PVA is of great interest to solve environmental issues nowadays. Properties such as biocompatibility, semi-crystalline nature, environmentally friendly, non-toxicity, high solubility in water, inexpensive, excellent film-forming capacity, excellent tensile strength, high dielectric constant and high thermal stability (200 °C) makes PVA a prospective material for the PEs [40]. The utilization of PVA as a substantive material as a host polymer for electrolytes are reported earlier [41].

### 5.2. Physicochemical Properties of PVA

The physicochemical properties of PVA depend mostly on its molecular weight and the proportion of hydrolysis. Depending on the length of the initial vinyl acetate polymer, the degree of hydrolysis to remove the acetate groups, and whether it occurs under alkaline or acidic conditions, the molecular weights achieved for PVA products can vary from 20,000 to 400,000 [50]. PVA possesses strong tensile properties, increased flexibility, hardness, and features that act as a barrier for gases and aroma characteristics. PVA has significantly better characteristics as an oxygen barrier than any other polymer that is currently known; nonetheless, moisture must be avoided in order to prevent the loss of its permeability toward gas [51]. The degree of hydrolysis, molecular weight, and crystallization of PVA all have a significant impact on the substance’s water solubility and physical characteristics, particularly its film form [52]. When PVA is formed, it is partially crystalline and has characteristics including chemical resistance, water solubility, and biodegradability. It is compatible with human tissues because of the similarity in physical characteristics. PVA membranes have been extensively developed for use in biomedical applications because biocompatible PVA has a structure that can absorb protein molecules, can engage with low cell adhesion, and is toxin-free. PVA can be physically or chemically bonded with other polymers to form composites. Due to their exceptional chemical and physical properties, biocompatibility, stability and non-toxicity, PVAs are very common polymers that are widely used as surface materials, in electrolytes, cement, medicals, glues and many other applications and glues [50,53]. Figure 10f presents the chemical structures of PVA. The degree of hydrolysis, specifically, whether it is complete or partial, determines the characteristics of PVA.

### 5.3. Why PVA-Based SPEs

There are two main groups of polymers: natural and synthetic biopolymers. Different suitable and renewable biopolymers such as cellulose, starch, PVA, chitosan, and others were used as host polymers. Previous studies have reported that PVA is one of the attractive polymers for the synthesis of PEs. PVA is a hydrophilic polymer and features a high density of reactive chemical functional groups. This functional group are substantial for mixing with conducting salt and a plasticizer [54].

PVA was reported to retain some essential features such as ease of preparation. Excellent charge storage capacity makes PVA a capable candidate for polymer electrolytes (PEs) for application in flexible electrochemical devices, especially EDLC [55]. However, the major challenge of using PVA alone as an electrolyte material or membrane is that they suffer stability issues over long cycles. Mechanical stress due to the volumetric change during charge–discharge over long cycles can lead to cracks in the membrane. These issues can eventually cause a capacity drop or breakdown over time. To increase the life cycle of a polymer-based supercapacitor is to incorporate the polymers with other forms of materials (e.g., salts, Al_2_O_3_, SiO_2_ and other materials) [56,57]. Therefore, PVA is used as a membrane in many applications, for example, as inorganic separations, etc. [40]. To improve the performance and stability of PVA membranes, several chemical modifications have been employed, e.g., cross-linking [58], solution casting [59], electrospinning [60], blending [61], sol-gel process [12], dip coating [62] and grafting [63].

### 5.4. Roles of Salt in PVA-Based SPEs

The properties of PEs have been found to depend upon the structure of the polymer network that makes up the electrolyte as well as on the interaction of the network and the salt. As the polymer networks are solvated by a large amount of the trapped solvent, electrolytes generally possess high mobility [64]. The polymer electrolyte is usually formed by adding the polymer to the solvent–salt solution. The ionic conductivity of PE generally depends on the dielectric constant of the host polymer and the lattice energy of the conducting salts [26]. In polymer electrolytes, the salt generally provides free/mobile ions that take part in the conduction process, and the solvent helps in solvating the salt and acts as a conducting medium, whereas the polymer is reported to provide mechanical stability by increasing the viscosity of the electrolyte. The salt used should generally have large anions and a low dissociation energy so that it easily dissociates. The salts should have also bulk anions. The solvent used should have a high dielectric constant, low viscosity, high boiling point, low melting point and low molecular weight. Similarly, within the framework, the salts in PEs serve as the sources of the charge carriers, which are generally required to have large anions and low dissociation energy for easier dissociating-induced free/mobile ions. In addition, for a polymer material to be used as a polymer host in PEs, it must possess the following properties: (i) fast segmental motion of the polymer chain; (ii) special groups promoting the dissolution of salts; (iii) low glass transition temperature (T_g_); (iv) high molecular weight; (v) wide electrochemical window; (vi) high degradation temperature [65].

**Figure 11:**
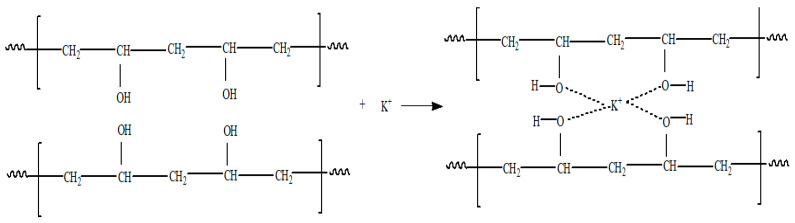
Possible structure for the synthesized PVA-K_2_CO_3_ composite electrolyte [66].

The previous literature reveals that electrical transport properties in a polymer occur through the amorphous phase rather than crystalline. In view of this concept, it appears more appropriate to select a polymer host that is predominantly amorphous, such as PVA, which has a very high amorphous content at room temperature [17]. In addition, PVA is a lightweight and transparent polymer that has a lower reactivity toward the lithium-metal-based anode, thereby providing scope for the improvement of electrode–electrolyte interfacial stability. These expected advantages provided substantial motivation to modify PVA, which is predominantly insulating with only electronic transport, into an ionically conducting system. In addition to combined high mechanical strength and temperature resistance, the O–H groups in PVA, a source of H-bonding, are capable of constructing polymer complexes as shown in Figure 11, where PVA forms a complex with K_2_CO_3_ salt [66].

The dissolution of the salt in PVA is easy due to the presence of polar groups (–OH) and high chain flexibility. Due to the high value of the dissociation constant of the polymer, PVA is fully dissociated in the electrolyte and hence provides a large number of H^+^ ions or conduction [67]. Subsequently, a number of ionically conducting PE based on PVA with different combinations of salts and solvents, such as PVA-NH_4_I [68], PVA-NH_4_Br [68], PVA-NH_4_Cl [68], PVA-MgNO_3_ [39], PVA-NH_4_NO_3_ [69], PVA-LiOH [70] and PVA-K_2_CO_3_ [66] with solvents such as acetonitrile, ethylene carbonate, distilled water and propylene carbonate, etc., have been reported in the literature. Furthermore, most of the conducting salts used in PEs possess a very high lattice energy. The high lattice energy salt was reported to have low ionic conductivity, as the movement of ions is highly restricted due to the presence of cohesive energy that will not allow the free movement of ions in the electrolyte. Salt concentration plays a crucial role in controlling the conductivity level and determining the nature of the interaction of the ions with the polymer matrix [65].

## 6. Formation of PVA-Based Polymer Electrolyte and Complex Formation

Figure 8 presents a possible structure for the synthesized PVA-K_2_CO_3_ composite electrolyte as reported by Bashir and co-authors [66]. The authors prepared the electrolyte by incorporating conducting salt (K_2_CO_3_) into the polymer host (PVA). The electrolytes were formed from the PVA hydroxyl or polar groups (-OH) bonded to the K_2_CO_3_ cations through a covalent dative bond. This is because polymer materials such as PVA can react with different organic or inorganic salts via -OH groups from its macromolecular chain and three-dimensional networks. The presence of polar –OH groups in the PVA permits chemical interactions (complex reactions) and physical interactions (either by H bonding, van der Waals dipole-ion or dipole–dipole interactions) [71]. As they reported, the K_2_CO_3_ dissociates into cations (K^+^) and anions (CO_3_^2−^) upon interaction with the solvent (dissolution) as shown in Equation (2).

(2) $$K_{2}{\mathrm{CO}}_{3\;\left(s\right)}\to {\;K}_{\left(\mathrm{aq}\right)}^{+}+{\;\mathrm{CO}}_{3\;\left(\mathrm{aq}\right)}^{2-}$$

The cations formed coordinates with the polar groups (-OH) from the polymer matrix and formed a complex (composites) compound. The linkage between the polymer polar groups and the cations from the salts (K^+^) generates a higher number of ion-conducting sites and a better interfacial interaction, thus resulting in an increase in the electrolyte’s ionic conductivity [72]. The interactions between metal ions from the salts with polar groups (-OH) of the polymers result mainly from electrostatic forces and accordingly lead to the formation of coordinating bonds. There are some important factors that may have an effect on the polymer–metal ion interactions, such as the nature of the functional groups attached to the polymer backbone, compositions and distance between functional groups, molecular weight, degree of branching, nature and charge of metal cation, and counter ions [65]. The cations from the salts can transfer from one coordinated site to another when subjected to an electric field. This is due to the weak coordinate of the cations to sites along the polymer chain [73]. It was reported that ions, mostly cations which are interconnected with functional groups of the host polymer chains, can move through re-coordination along the polymer backbone. Based on this, the polymer chains are folded to form tunnels, in which the cations are located and coordinated by the functional groups. These tunnels create channels, providing a pathway for the movement of cations [74].

**Figure 12:**
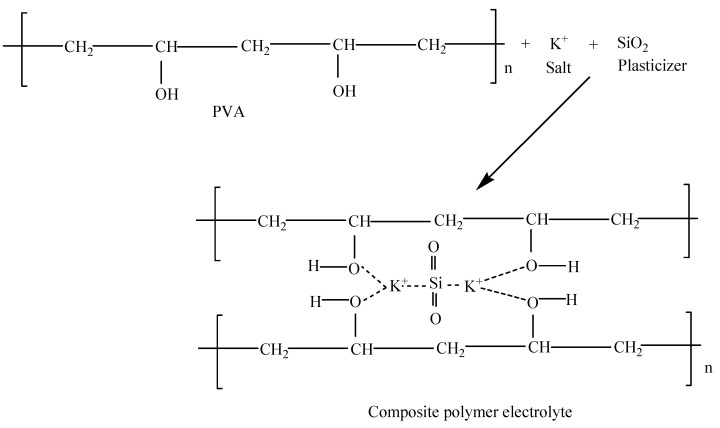
Possible structure of the synthesized PVA-K_2_CO_3_-SiO_2_ composite PE [75].

The complex formation between PVA, salt and filler (SiO_2_) were reported earlier [75]. The electrolyte was prepared by incorporating conducting salt (K_2_CO_3_) and the filler into the polymer host (PVA). Upon dissociation of the conducting salt into cations and anions, the K^+^ chemically coordinated with the polar (-OH) groups of the PVA in the presence of SiO_2_. This coordination results in the formation of weak bonds between the hydroxyl group of PVA, K^+^ and SiO_2_, which results in a complex formation as shown in Figure 12 [76].

In addition, the partial or weak bond formed between K^+^ and SiO_2_ with the polar groups of the polymer could aid in hastening the conduction of ions since the ions are in a moveable state [56]. Similarly, silica material was found to affect the changes in the morphological structure of the electrolytes [77]. The silica external surface may chemically coordinate with the ions and subsequently deliver extra sites generating favorable tunnels or ways for ion transport within the locality of the electrolytes [76]. The chemical coordination within the electrolytes might increase the conduction sites for the movement of ions, thus creating extra conducting ways for the migration of ions [78].

### Charge Carriers (Mechanism) and Ion Mobility

**Figure 13:**
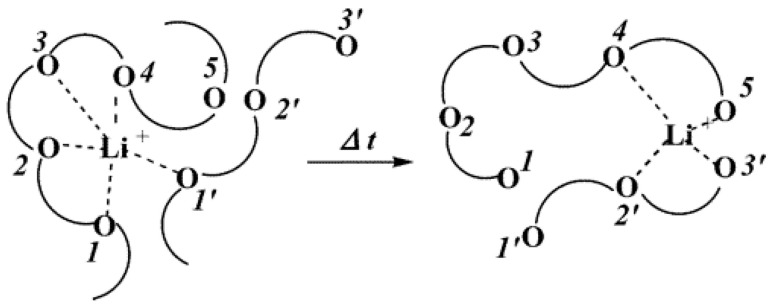
Schematic diagram of lithium-ion conduction mechanism of polymer-based polymer electrolyte [80].

In order to develop SPEs with improved ionic conductivity, the polymer should not only dissolve the conducting salt but also be able to couple with ions in the salt. The polar groups in the polymer such as —OH should be effective building blocks for dissolving any conducting salts. The major research on all-SPEs nowadays is focused on PVA and other polymers and their derivatives. This is because the polar group in the PVA is coordinated with the ion in the salt through Coulombic interaction, initiating the anion and cation of the salts to dissociate. When an electric field is applied, the movement of cations and anions from one coordination point to another along the polymer segments from one segment to another will initiate [79]. A previous study has reported the ion transport mechanism of polymer electrolytes using Li^+^ as a conducting ion, as shown in Figure 13 [80].

In the polymer–salt complex system, ions are not free to move due to the huge size of the polymer chain in addition to the boundary effect of crystalline domains. The factors affecting ionic conductivity are the number of ions and the mobility of the polymer chain. The amount of ions moved will be influenced by the ability of the polymer to dissociate ions in the salt, and thus, the salt of low lattice energy and the polymer of high dielectric constant can promote this dissociation [81]. The ionic concentration of an electrolyte was reported to be influenced by the duo of lattice energy and the dielectric constant of the conducting salt and the host polymers, respectively. The lower the lattice energy of the conducting salt and the higher the dielectric constant of the host polymers, the higher the ionic concentration. Under steady-state conditions, the ionic conductivity, *σ*, of an electrolyte can be given as in Equation (3) [65]:

(3) $$\sigma \;=\;\underset{i}{\sum }\eta_{i}q_{i}\mu_{i}$$

where *η_i_* is the concentration of charge carriers, *q_i_* is the charge on electron and is the mobility of ions, and *i* refers to the type of the ions. From Equation (3), the ionic conductivity (*σ*) can be increased by increasing the concentration of charge carrier (*η_i_*) or the ionic mobility in the system. It was reported that the charge density, *η*, depends largely on both dielectric constant (*ε*′) and dissociation energy (*U*) of the polymer as given in Equation (4) [82]:

(4) $$\eta =\eta_{o}\exp(-U/\varepsilon \prime k_{B}T\;)$$

where *k_B_* is the Boltzmann constant and *T* is the absolute temperature. However, owing to the link between dielectric constant (*ε*′) and charge carriers, an increase in *ε*′ is the same as an increase in charge carrier concentrations. This is because *ε*′ is linked to the ratio of the capacitance (*C*) to the capacitance of the cell (*C_o_*) (*ε*′ = *C/C_o_*), whereas capacitance is associated with the amount of stored charge (*C = Q/V*) in which *Q* is the total charge and *V* is applied voltage [82]. It can be observed from Equation (4) that the concentration of charge carrier (*η*) can be increased by the increase in *ε*′ [83]. Therefore, it can be seen that in the polymer electrolyte, the increment of the concentration of the movable ions and the migration speed of the ions can increase the conductivity of the ions.

**Figure 14:**
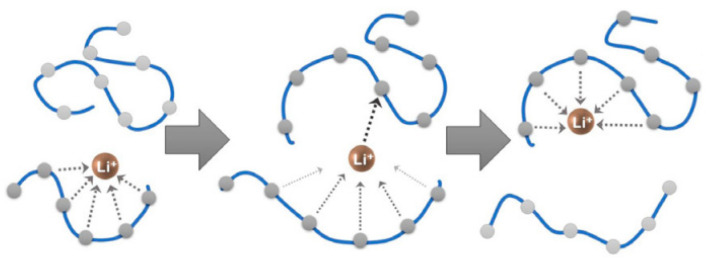
Ion transport by hopping and segmental motion in SPE [85].

The movement of mobile charge carriers (or ions) which dissociate from the complexes causes ionic hopping in polymer electrolytes. Some basic conditions must be satisfied to create the mechanism for ionic conduction through hopping, such as the availability of mobile ions for migration in addition to the availability of vacant sites for the ions to occupy. In addition, for good conduction to happen, the vacant and involved destinations of the electrolyte should have equivalent potential energies and a low activation energy boundary for bouncing instigated by thermal vibrations. Low activation energy for bond breaking is fundamental for a high bouncing rate since particle transport in electrolytes is a thermally activated jumping process [49,84]. A frequently reported mechanism is portrayed for Li^+^ ion transport in ether-based SPE in Figure 14 [77]. It observed that the ionic conduction process is linked to either the main chain segmental motion or Li^+^ ion hopping process for polyether-based SPEs [85].

**Figure 15:**
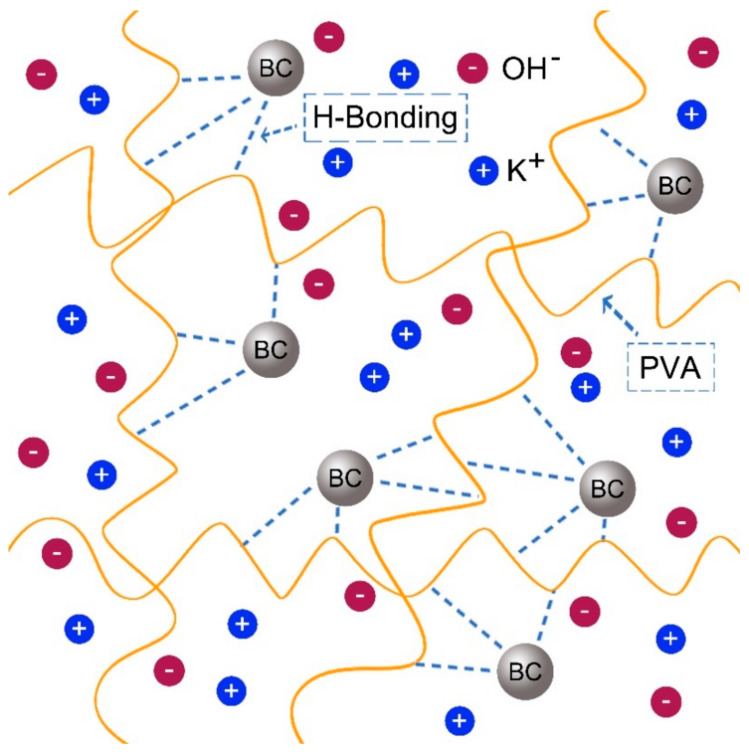
The schematic diagram of the PVA-BC-KOH electrolyte membrane structure [54].

Consequently, Polu and R. Kumar has reported that the conductivity of PVA-based SPE is generally initiated by two conductive mechanisms: the transfer of ions along the polymer molecular chain through the dissociation and combination process between ions and −OH of the polymer matrix [39]. Another major factor is the conducting salt providing more ion transfer channels due to the swelling structure where the movement of ions increased at higher content [41]. The two mechanisms depend on the crystalline-to-amorphous ratio, which might be decreased by the addition of the conducting salt. The increase in amorphous region and free volume could support local PVA chain segmental motion. Hence, the ions migration increased at a higher salt amount where more ions will migrate at the same time, and the conductivity improved. There are many hydroxyl groups that exist on the surface of the PVA chain, so the hydrogen bond should be easily formed. The proposed schematic structure and ion movement model of PVA-salt SPE is shown in Figure 15 [54].

## 7. Electrochemical Performances of PVA-Based Polymer Electrolytes

There are two important electrochemical characterizations used to measure electrochemical performances and permittivity of electrolyte materials as functions of frequency at different temperatures. One type of characterization is electrochemical impedance spectroscopy (EIS) which determines the electrochemical behavior (ionic conductivity) of an electrolyte. The other type is linear sweep voltammetry (LSV), which measures the cell voltage or stability window of an electrolyte material. These characterizations offer an in-depth understanding of the structural and electrical properties of the electrolyte.

### 7.1. Electrochemical Impedance Spectroscopy (EIS)

EIS is a powerful analytical tool used to characterize the electrical and ionic properties of PEs and their interfaces. It was reported that the ionic conductivity of polymer-based ionic conductors mainly depends on the concentration of charge carriers and their mobility. Hence, impedance spectroscopy has been found to be a simple and powerful method to study the electrical and ionic properties of polymer-based electrolytes. The displacements of electrical charge in an electrolyte material were found to produce two separate physical phenomena. (1) If the charge motion is in a restricted volume of the matter and is strictly confined, then a polarization phenomenon would take place and (2) when the electrical charge in the electrolyte materials diffused jointly, then diffusion is possible and ionic conductivity is established [86].

To identify and overcome the effect of space charge polarization at the electrode/electrolyte interface, it is critical to carry out the conductivity measurements by means of EIS using an electric field. It is well known that spectroscopy depends on the tendency of ions and dipoles to orient along the electric field direction [65]. When an electric field is applied to a parallel plate of a capacitor sandwich with electrolyte materials, different groups of polarization are taking place, which are known as electronic, atomic, dipolar and migrating charge polarizations. Dipolar polarization and polarization due to charge migration are the two main components of the dielectric responses in PEs that can be detected at a certain frequency normally less than 109 Hz. However, if the electric field is reversed in sign (or direction), the dipoles will be realigned with the applied field and the ions start to migrate to the other side of the electrode. As the frequency of field reversal increases, the ions and dipoles become increasingly difficult to keep up with the field changes that lead to low ionic conductivity [84].

### 7.2. Principle of Electrochemical Impedance Spectroscopy

EIS is based on a simple principle when a sinusoidal potential (a sine wave input) is applied across the sample whose impedance is to be measured. For a linear system, the input and response of AC signal are expressed as in Equations (5) and (6) [87].

(5) $$V\left(t\right)=\;V_{0}\sin\;wt$$

(6) $$I\left(t\right)=\;I_{0}\sin\left(wt+\varphi \right)$$

where *V*_0_ and *I*_0_ are the maximum voltage and current, *ω* and *φ* represent the angular frequency and phase angle, respectively. Then, the impedance parameter is defined as *Z(ω)*, which defines the ratio between the applied voltage and the resulting electric current, *Z(ω)* = *V(t)/I(t),* and express as in Equation (7):

(7) $$Z\;=\;Z_{real}^{\prime }+\;\;jZ_{imag}^{\prime \prime }$$

where *Z*′ and *Z*″ represent the real part and imaginary part of the impedance, respectively, and can be determined at a given frequency by Equations (8) and (9) [65].

(8) $$Z_{real}^{\prime }\;=\;\left|Z\right|\cos\theta$$

(9) $$Z_{imag}^{\prime \prime }\;=\;\left|Z\right|\sin\theta$$

Following EIS technique, the real (*Z_real_*) and imaginary (*Z_imag_*) parts of the impedance can be obtained over a varied range of frequencies where the impedance experimental data can be analyzed by plotting the imaginary part *Z_imag_* versus the real part *Z_real_* and hence information regarding equivalent circuit can be extracted. The intercept of the impedance spectrum at the real axis gives rise to the bulk (ionic) resistance (*R_b_*) of the electrolytes from which ionic conductivity will be calculated following Equation (10) [84].

(10) $$\sigma =\;\;\frac{t}{R_{b}A}$$

where *t* is the thickness of the samples (cm) and *A* is the electrode–electrolyte contact area (cm^2^) [65,84].

### 7.3. Impedance Spectroscopic Analysis

**Figure 16:**
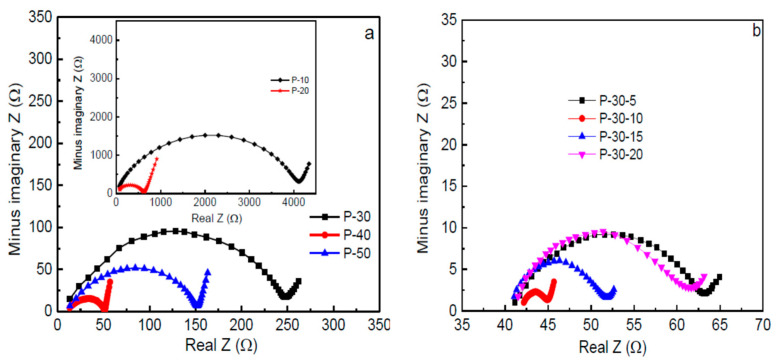
Cole–Cole plots of (**a**) P-10, P-20, P-30, P-40 and P-50 films; and (**b**) P-40-5, P-40-10, P-40-15, and P-40-20 films [88].

The basis of impedance spectroscopy is to investigate and study the bulk and interfacial resistances of any electrolyte where the R_b_ will be determined for ionic conductivity study. Hence, many authors reported the impedance analysis. For instance, the impedance spectroscopic of PVA-based SPEs was studied by Wang and co-authors [88]. The Nyquist impedance plots of PVA-based electrolytes incorporated with LiTFSI in different wt.% and PVA incorporated with 40LiTFSI + EMITFSI in different wt.% are presented in Figure 16a,b, respectively. All the samples displayed a characteristic impedance pattern of polymer electrolyte, with a single semi-circular curve at high frequencies, and a spiked line at low frequencies [89]. Apparently, the circular arc gradually shrinks with increasing LiTFSI content up to 40 wt.% and then enlarges, which signifies that the bulk resistance of the electrolytes decreases and increases, suggesting an increase or decrease in ionic conductivity of the electrolytes. This characteristic behavior was ascribed to the presence of the high amount of mobile Li^+^ ions that are available due to the presence of a high content of conducting salts to coordinate with PVA and jump from one side of the polymer matrix to another. It was further found that with the incorporation of EMITFSI-conducting salts, the circular arc continues to shrink, owing to the dissolution of the salt in the PVA matrix, which largely acts as a plasticizer that makes the polymer chains more flexible and the amorphous domains expand, resulting in an increase in ion mobility *µ_i_* [88].

**Figure 17:**
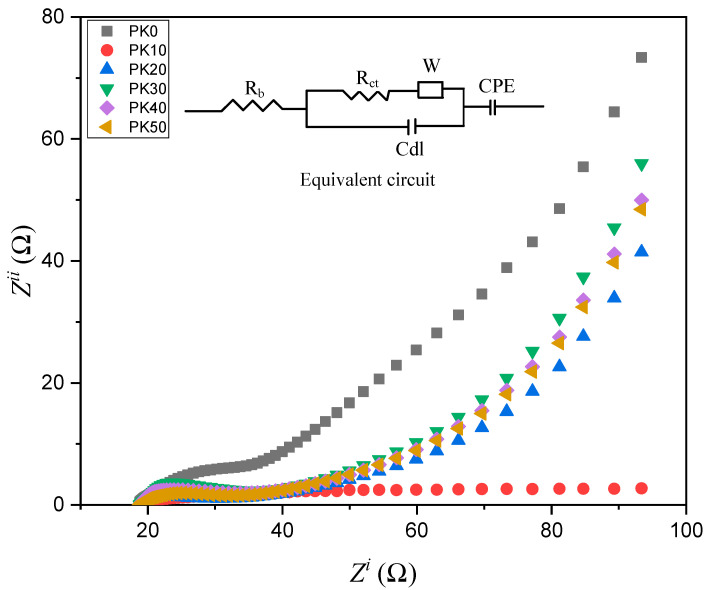
Cole–Cole plot for pure PVA and PVA-K_2_CO_3_ SPEs [66].

Further, the Nyquist impedance plots of PVA and PVA-K_2_CO_3_ composites is presented in Figure 17, as reported by Bashir and co-authors [66]. The impedance patterns displayed a single semi-circular arc at high frequencies and an inclined line at low frequencies. The R_b_ gradually continue to decrease with increasing K_2_CO_3_ content up to 30 wt.% and then increases, implying that the ionic conductivity increases and then decreases. This is ascribed to the greater quantity of mobile K^+^ ions that are available in the 30 wt.% PVA-K_2_CO_3_ composites to coordinate with PVA material which makes the polymer chains more flexible and the amorphous domains, resulting in an increase in ion mobility *µi*.

In order to provide information about the interface properties of the synthesized electrolytes, the authors proposed an equivalent circuit of the Cole–Cole plots shown in the inset of Figure 17. The semicircle region from the EIS result is constructed of a parallel combination of a capacitor and resistor, as represented in the equivalent circuit model. The bulk properties (*R_b_*) of the electrolytes refer to the capacitance and interfacial contact between the electrodes [62]. The capacitor is denoted by the capacitance of double layer (*C_dl_*), which comes from the formation of an electrical double layer owing to the accumulation of charge carrier and ions at the electrode and electrolyte interface after the ions diffused into the porous electrode and subsequently deliver high ionic conductivity. The C_dl_ was connected in parallel with charge transfer resistance (*R_ct_*) and capacitance that are included in the series in order to evaluate the contribution of pseudocapacitance. Thus, the results have established that the successful incorporation of the conducting salt (K_2_CO_3_) into the polymer matrix can improve the diffusion of ions in the electrolyte and consequently promote the ion adsorption at the electrode/electrolyte interface that leads to an increase in ionic conductivity [9]. The resistance *R_ct_* is denoted to represent the charge transfer resistance of the electrolyte on the charge absorption across the electrode/electrolyte interfaces. Hence, the ions must overcome the resistance to form the electrical double layer. The total internal resistance of the cell, which is the combination of R_b_ and R_ct_ was computed from the intercept of the semicircle [62].

**Figure 18:**
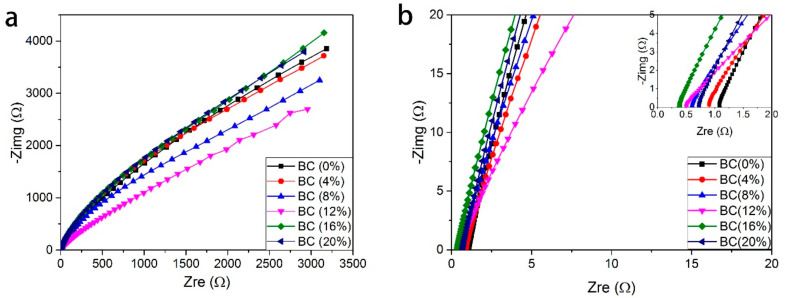
The impedance spectra of the PVA-BC-KOH electrolyte membranes with different contents of BC filler; the inset of b for the high-frequency area [54].

Similarly, Fan and co-authors reported SPEs based on PVA Alkaline incorporated with Bamboo Charcoal (BC) [54]. The studies show typical AC impedance spectra for the prepared electrolytes-based PVA incorporated with BC and KOH as shown in Figure 18a,b, (**Figure 18b showed the magnify version of Figure 18a for clear vison**). It was clearly shown that the typical EDLC impedance plots are observed for both samples. The observed spectra consist of a typical spike in both frequencies. The spike observed can be attributed to the double-layer nature of the interface between the electrode and electrolyte in the high-frequency range [90]. The bulk resistance was taken at the intercept of the Cole–Cole plot with the real axis. It was found that the bulk resistance decreases with the increase in BC concentration [54].

### 7.4. Ionic Conductivity

The ionic conductivity of a polymer electrolyte is crucial to determine the electrochemical performance of a supercapacitor device. The ionic conductivity was reported to effectively affect the movement of ions between two electrodes, and for large ionic conductivity electrolytes, the movement of ions within two electrodes is very fast and definitely helps in improving the power and energy density of the supercapacitor [66]. Similarly, the electrochemical performance of PEs primarily relies upon (a) crystallinity of the host polymer, (b) instantaneous motion of anion and cation, and (c) ion-pair formation. The conductivity of PEs is low when compared with the present conventional liquid electrolytes [25].

**Figure 19:**
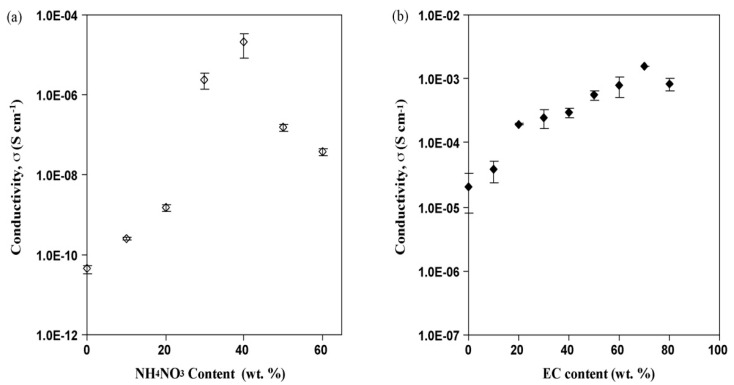
Variation of conductivity of chitosan–PVA on (**a**) NH_4_NO_3_ and (**b**) EC concentrations at room temperature [69].

Kadir and co-authors studied the effect of salt content on the ionic conductivity of the PVA–chitosan electrolyte [69]. The authors reported that the conductivity of the pure chitosan–PVA blend is low at room temperature, as presented in Figure 19. From the figure, the authors observed a significant increase in the conductivity of chitosan–PVA with the incorporation of NH_4_NO_3_ and ethylene carbonate (EC) concentration. The authors attributed the increase in conductivity to the increase in salt concentrations, which can dissociate and provide more ions that contributed to the increase in conductivity. The study reported that, at a higher salt concentration in the blend, the rate of ion dissociation is observed to be higher than the rate of association. However, they observed that the ionic conductivity increased progressively with the increase in salt concentration until 40 wt.%. Beyond 40 wt.%, the conductivity decreases, and this may be connected to the distance between dissociated ions becoming too close, such that they are able to recombine and form neutral ion pairs that do not contribute toward conductivity [91]. Thus, the number density of ions in the polymer matrix decreases, which resulted in a decrease in conductivity. Similarly, the mobility of the ions was found to decrease at a higher salt content as the increase in salt concentration will increase the viscosity of the solution [92].

**Figure 20:**
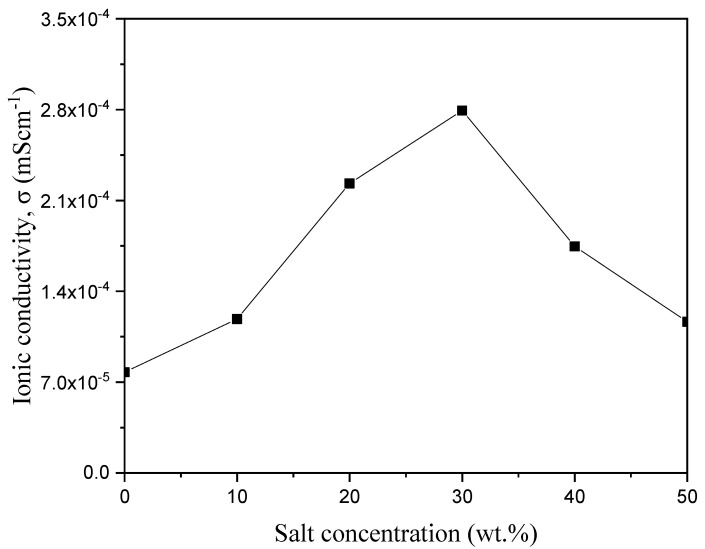
Effect of salt concentration on the ionic conductivity of PVA-K_2_CO_3_ at ambient temperature [66].

In their work, Bashir and co-authors reported the ionic conductivity of the synthesized polymer electrolyte based on PVA incorporated with K_2_CO_3_ composites, and the result is shown in Figure 20 [66]. From the figure, the ionic conductivity of the electrolyte was reported to increase with an increase in K_2_CO_3_, where the ionic conductivity attained was at a maximum at 30 wt.%, then slowly decreased with the increase in wt.% amount confirming that the ionic conductivity of the electrolyte improved with the appropriate doping amount of K_2_CO_3_. This is due to the amount and mobility of mobile charge carriers that have reached the optimum level in this conductive polymer electrolyte. Furthermore, the strong conducting effect of K_2_CO_3_ aids the softening of the PVA backbone and hence increases the flexibility of the polymer chain. The ions can be transported easily within the polymer matrix with highly flexible polymer chains. Moreover, the higher flexibility of polymer chains is reported to improve the mobility of polymer segments and assists the ionic transport in the polymer complexes [93]. Likewise, a conducting salt such as K_2_CO_3_ can decrease the *T_g_* of polymer electrolytes [66].

**Figure 21:**
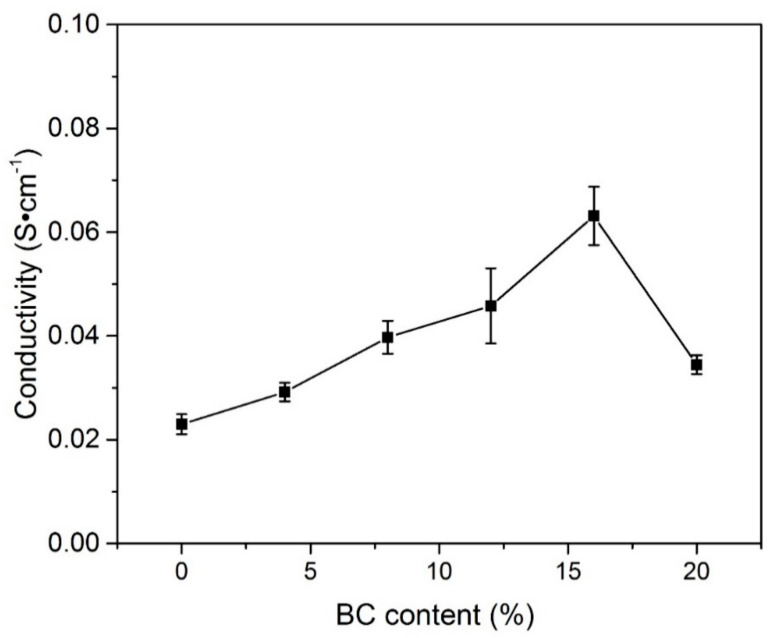
The conductivity of the PVA-based electrolytes with different amounts of BC [54].

Likewise, Fan and co-authors studied and reported the results of ionic conductivity obtained through Equation (10) for all PVA-BC-KOH PEs with different amount of BC. The authors observed that when the amount of BC is lower than 16 wt %, the resultant ionic conductivity value increased as presented in Figure 21. It was established that the ionic conductivity greatly depends on the amount of BC added, where the highest ionic conductivity was reached when the BC amount is 16 wt %. However, the ionic conductivity decreases when the amount of BC goes beyond the optimum (16 wt %) [54].

Subsequently, a number of ionically conducting polymer electrolytes based on PVA with different combinations of salts, such as NH_4_I [68], NH_4_Br [68], NH_4_Cl [68], MgNO_3_ [39], NH_4_NO_3_ [69], LiOH [70] etc., have been reported in the literature, as summarized in Table 1.

### 7.5. Potential Window

Linear sweep voltammetry (LSV) is an electroanalytical chemistry applied to study the electrochemical stability window (ESW) of SCs or potential energy pioneered by Heyrovsky, in 1959 [105]. The experimental system for LSV utilizes a potentiostat in a three- or two-electrode setup to deliver a potential to a solution and monitor its change in current. The electrode setup usually consists of a working electrode, a counter electrode, and a reference electrode. The potentiostat brings the potentials over the electrode setup. A potential, E, is delivered through the working electrode. The slope of the potential against time graph is called the scan rate and can range from mV/s to 1,000,000 V/s. The working electrode is one of the electrodes at which the redox reactions occur, and the processes that happen at this electrode are the ones being observed. The auxiliary counter electrode is the one at which a process opposite from the one taking place at the working electrode occurs, which is not monitored [106,107].

**Figure 22:**
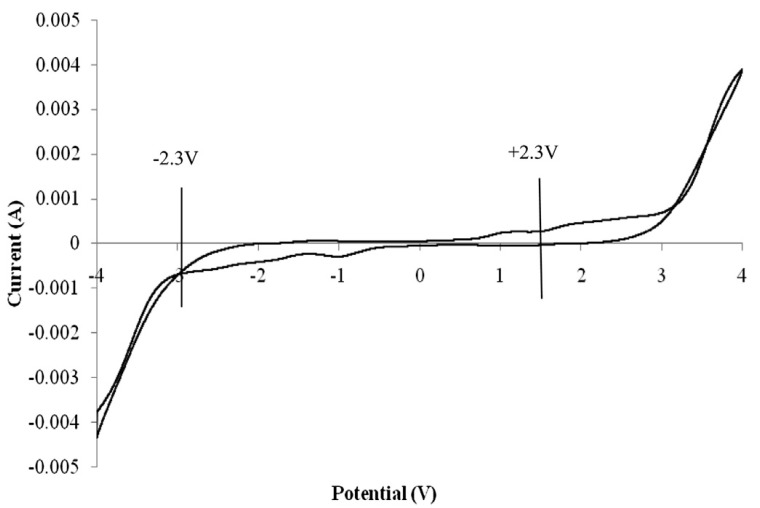
ESW for EDLC cell with PVA-LiCLO_4_-TiO_2_ electrolyte [90].

The ESW is the main parameter to be examined in an electrochemical device such as EDLC that explains the maximum operational potential of energy stored in the device. In their study, the performance of a polymer electrolyte based on PVA incorporated with LiCLO_4_-TiO_2_ in terms of electrochemical behavior, the charge storage at separate interfaces in the anodic and cathodic regions was evaluated and reported by Lim and co-authors, and the result is presented in Figure 22. The results designate the ESW results at a scan rate of 10 mVs^−1^. From Figure 22, the prepared electrolytes based on PVA-LiCLO_4_-TiO_2_ show highly stable potential energy where the electrolytes do not undergo any chemical transformation within the potential region [90].

**Figure 23:**
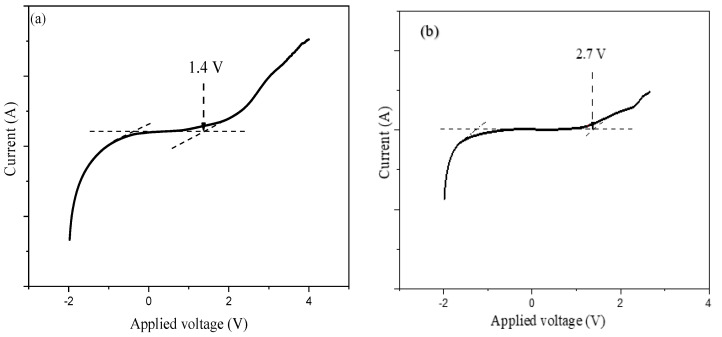
LSV plots of the synthesized PE PK0 (pure PVA) and PVA-K_2_CO_3_ composites [66].

Similarly, the ESW of the PEs based on PVA-K_2_CO_3_ was studied using LSV and reported by Bashir and co-authors, and the result is depicted in Figure 23 (and b) [66]. The authors found that the current remains nearly continuous with increasing applied voltage until the potential reaches a certain limit (V_max_), which subsequently increases. The sudden increase in the current has been considered as the upper limit of the electrolyte’s stability range. The results show that no electrochemical decomposition occurs when the voltages are below 1.4 V for all the electrolytes, which are wide enough to enable safe application in any energy storage devices such as EDLC **(as shown in Figure 23a)**. The range of electrochemical stable windows increases with increasing the K_2_CO_3_ content until 30 wt.% **and reached 2.7 V (as shown in Figure 23b)**. The synthesized PEs in this study have a substantial value of potential window that is a sufficient working voltage range for supercapacitor applications. As the authors reported, the improved electrochemical windows of the prepared PEs could be attributed to the unique properties of the conducting salts used [108].

Furthermore, the study reported that pure PVA shows a slightly lower potential window compared with PVA-K_2_CO_3_ indicating that the addition of K_2_CO_3_ improved the electrochemical oxidation stability [109]. The enhanced electrochemical stabilities may be due to the fact that the addition of K_2_CO_3_ increased the concentration of K^+^ and promoted the formation of a stable passive layer over the electrode interface [105]. Additionally, the incorporation of conducting salts into the polymer matric is known to increase the anodic and cathodic stability of the polymer electrolytes. Hence, this could be another reason for the increase in the potential window with the addition of K_2_CO_3_ [66].

**Figure 24:**
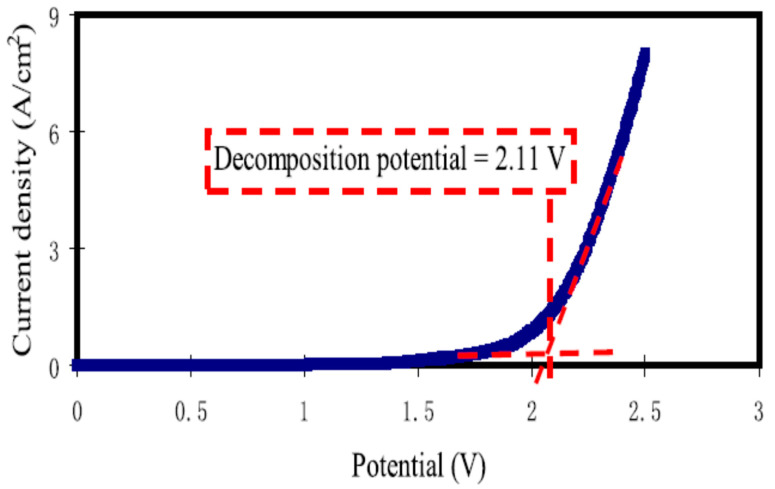
LSV for the PVA-based electrolyte [103].

Similarly, the structural, ion transport and electrochemical properties of polymer electrolytes based on PVA for EDLC applications were reported earlier by Srivastava and Varshney [103]. As shown in Figure 24, the ESW at 10 mV s^−1^ with a voltage ranging from 0 to 2.5 V was studied, and the potential for the developed electrolyte was to be suitable for EDLC application.

**Figure 25:**
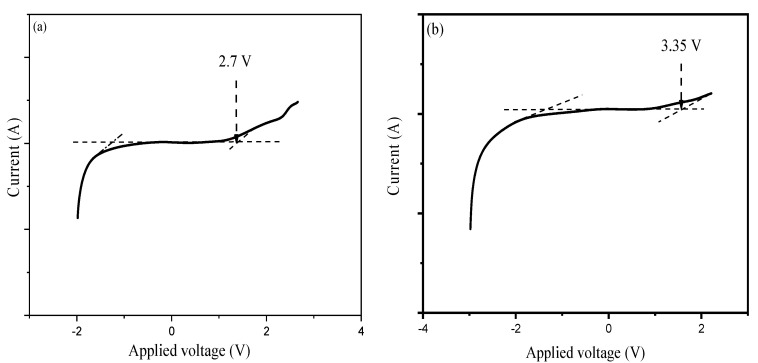
LSV plots of the synthesized PVA (**a**) un-plasticized and (**b**) plasticized PEs [75].

In another study by Abdulkadir et al., the ESW of the polymer electrolyte based on plasticized PVA-K_2_CO_3_ was examined at a 5 mV s^−1^ scan rate was reported previously, and the result was depicted in Figure 25a,b [75]. The authors observed that the plasticized electrolyte exhibited a wider electrochemical stability window than un-plasticized electrolyte and the authors attributed this to the inclusion of silica particles that enhanced the electrochemical stability of the electrolyte. Consequently, the authors concluded that the addition of SiO_2_ to the PEs considerably increased the potential window of the synthesized electrolyte. According to a reported study, electrochemical stability may be influenced owing to the influence of the silica and the dielectric constant of the host polymer, which would give a higher concentration of charge carriers [110]. Previous study has confirmed that SiO_2_ is a known ingredient that increases the amorphous phase of the electrolyte, which is attributed to the creation of mobile defects as well as an increase in the flow of the material possibly at the interface region [75].

Generally, PVA-based SPEs were reported to have higher potential window values which are ascribed to the presence of polar groups that can easily absorb salts (ions) and form complexes. Higher potential window is required for high power density of SCs. From Table 2, it is noticed that PVA-based electrolytes show moderate potential window values that are suitable for application in energy storage devices.

### 7.6. Dielectric Studies

Based on the previous studies, the concentration of charge carriers and segmental mobility are not the only features governing the conductivity behavior of an electrolyte, the others being dielectric constant (*ε_r_*) and dielectric loss (*ε_i_*). It was found that the increase in ionic conductivity with an increase in conducting salt could be ascribed to the increase in the number of free mobile ions, whereas the decrease in ionic conductivity could be linked to the ion association that leads to the generation of more non-conductive neutral ion pairs. Similarly, the decrease in conductivity is related to the decrease in dielectric loss [113,114]. The ionic conductivity behavior of an electrolyte material can be studied by ε_r_ and εi. [115]. The ε_r_ represents the material’s stored charges and also indicates the capacitive nature of the electrolytes, while *ε_i_* stands for the loss of energy to move ions and align dipoles when the electric field polarity reverses rapidly. From the impedance measurement real and imaginary components, ε_r_ and ε_i_ were calculated [116].

**Figure 26:**
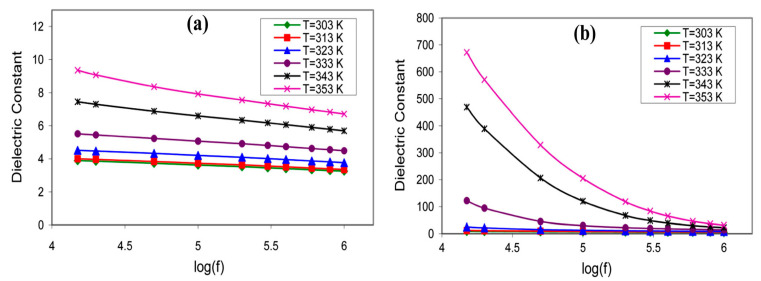
Dielectric constant as a function of frequency at various temperatures of (**a**) pure MC-PVA polymer blend and (**b**) MC-PVA doped with NH_4_NO_3_ [114].

Abdallah and co-authors reported dielectric studies of PVA-MC incorporated with NH_4_NO_3_, and the result is depicted in Figure 26 [114]. The result shows the variation of the dielectric constant as a function of frequency at different temperatures, for pure MC-PVA doped with NH_4_NO_3_ PEs, and the results show that the values of the dielectric constant are very high at low frequencies and high temperatures. The higher values of *ε_r_* at low frequencies might be associated with the charge accumulation at the electrode–electrolyte interface, and the permanent dipoles have enough time to rotate in the direction of the applied field [24]. It can be clearly seen that the dielectric constant values begin to drop after a certain critical frequency. This is due to the diffusion of ions and the dipole molecules, which cannot orient themselves in the direction of the applied electric field, at high frequencies [117].

**Figure 27:**
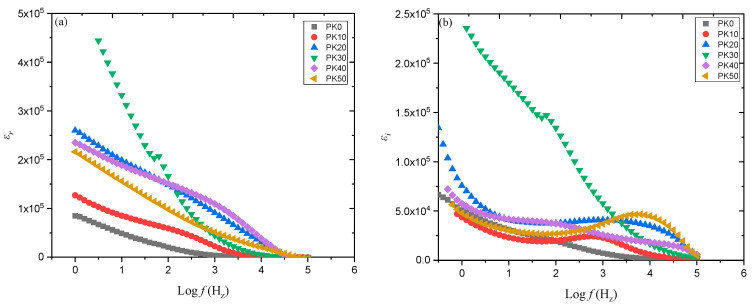
The plots of (**a**) *ε_r_* and (**b**) *ε_i_* against frequency for PVA-K_2_CO_3_ electrolytes [113].

Figure 27a,b shows the variations of *ε_r_* and *ε_i_* against the frequency for the synthesized PE based on PVA-K_2_CO_3_ with different weight percentages of K_2_CO_3_ at ambient temperature, as reported previously [113]. The study observed that there is a change in both ε_r_ and εi with a different weight percentage of K_2_CO_3_ with a change in frequencies. Both values are high and show dispersive behavior at low frequencies, owing to the polarization effect near the electrodes or the accumulation of the charge carriers [55,65,118].

## 8. Thermodynamic Effects and Ion Transport Models for Polymer Electrolytes

There are two important practical models affecting the thermodynamic transport and ion mobility mechanism in an electrolyte: the Vogel–Tammann–Fulcher (VTF) model and the Arrhenius model.

### 8.1. Vogel–Tammann–Fulcher (VTF) Model for Ion Transport

An important empirical model used to study the ion mobility in PEs is the Vogel–Tammann–Fulcher (VTF) model. In this model, a strong interrelation between conductivity and segmental relaxation in polymers is anticipated [65]. The non-linear Arrhenius plot of temperature-dependent conductivity data can be accurately described by the VTF Equation (11) as follows:

(11) $$\sigma \left(T\right)=\;AT^{-1/2}\exp\left[-\frac{B}{k_{B}\left(T-T_{0}\right)}\right]$$

where *A* is the pre-exponential factor related to the number of charge ions, *k_B_* is the Boltzmann constant, *B* is the pseudo-activation energy associated with the polymer segmental motion and *T_0_* (*T_0_* = *T_g_* − 50 K) is the temperature corresponding to zero configurational entropy and *T_g_* is the glass transition temperature. Previous studies ascribed the non-linearity behavior of ionic conductivity to the fact that the polymer segmental motion assists ion mobility. Based on the VTF model, the curvature behavior of the Arrhenius plots has been attributed to the presence of a strong interrelation between the ionic motion and polymer segmental relaxation. This implies that the polymer segmental relaxation and ionic motion are well combined with each other [65,119].

Therefore, based on this theory, it can be understood that the curvature behaviors of conductivity can be ascribed to the decoupling mechanisms between the ionic and polymer segmental motions. Hence, it can be concluded that from VTF models, the increase in ionic conductivity can be attributed to the hopping rate of the ions. In addition to these models, upon ion transport and conductivity behavior, ion dissociation energy and dielectric constant also have a great influence on the conductivity behavior of a polymer electrolyte.

### 8.2. Arrhenius Model for Ion Transport

Another important variable to study ion transport model in relation to temperature is the Arrhenius model. In this model, linear relations observed in electrolyte samples highlights that there is no significant phase transition in the synthesized PEs, i.e., the temperature dependence of ionic conductivity data can be accurately described by the Arrhenius Equation (12):

(12) $$\sigma \;=\;\sigma_{o}\exp\left[-\frac{E_{a}}{k_{B}T}\right]$$

where *σ_o_* is a pre-exponential factor, *Ea* is the activation energy and *T* is the temperature (K). The role of phase change from crystalline to amorphous phases of PEs on conductivity improvement in chitosan-based PEs was reported. The study of Arrhenius behavior of PEs based on PVA/PVdF:LiClO_4_ was reported previously [120]. The authors observed the polymer segmental motion assists the linearity behavior of the ionic conductivity showing the ion transport model. Based on the model, the linear behavior of the plot was attributed to the presence of a strong interrelation between the ionic motion and polymer segmental relaxation. This implies that the polymer segmental relaxation and ionic motion are highly dependent on each other.

**Figure 28:**
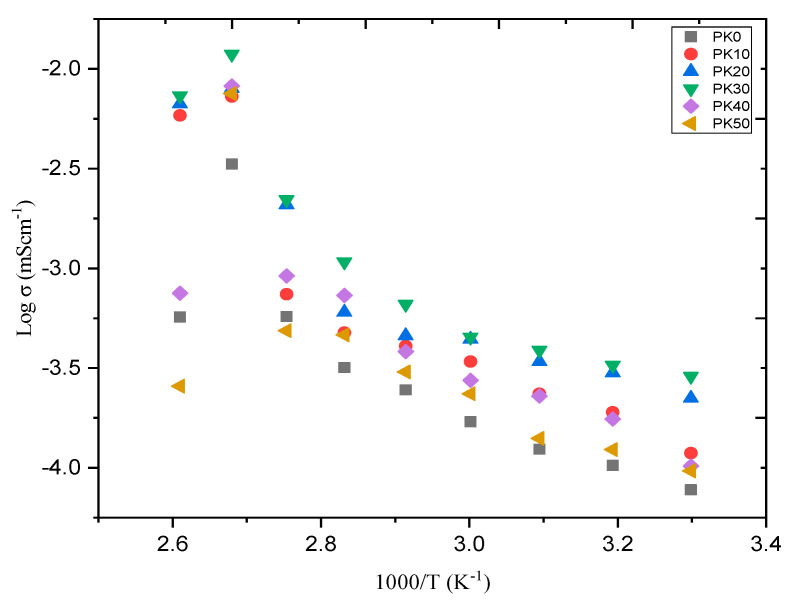
Conductivity versus temperature of PEs (PVA-K_2_CO_3_) electrolytes [66].

Figure 28 shows the temperature–conductivity relationship for PVA-based electrolytes incorporated with different amount of salt (K_2_CO_3_) and reported by Bashir and co-authors [66]. The authors reported that the conductivity increased linearly with increasing temperature following Arrhenius model, which could be attributed to the increasing movements of polymer chains and free K^+^ ions. At higher temperatures, the thermal movement of polymeric chain segments and salt dissociation could be improved. This encourages the fast movement of ions and consequently causes an increase in the ionic conductivity of the polymer–salt complex [121]. However, the study reported that the increase in ionic conductivity versus 1000/T is not a rapid process, which suggests that the jumping of mobile ions from one site to another is a thermally activated process [122].

## 9. Methods for Enhancing Performance of PVA-Based SPEs

Generally, SPEs have numerous advantages over liquid for application in supercapacitors. The key issues about SPEs are their low ionic conductivity and potential window that need to be improved [24]. The ultimate goal is to obtain SPE with a wider potential window, high ionic conductivity, good mechanical strength, higher operating temperature, and high compatibility with electrode materials to achieve higher performance. In order improve the ionic conductivity of the SPE, many methods have been adopted to modify and adjust the chemical structure of SPEs to improve the ionic conductivity. These approaches include modifications on the polymers, blending of different polymers, mixed salt systems, and additives commonly known as plasticizers/fillers [17,65,69].

### 9.1. Polymer Modifications

The conductivity of the SPEs was found to increase with the incorporation of conducting salts and plasticizers/fillers. The crystalline part of a polymer matrix hinders the ion-conducting path in the electrolytes. Consequently, there is a need to alter the surface of the polymer to increase the degree of amorphousness. Studies report that polymer electrolytes with salt as an additive have flexible chains that simplify the polymer segmental mobility that helps in transporting ions through the polymer matrix. The major aim of additives such as salts in SPEs is to improve the amorphous region in the host polymer via an ion transfer mechanism to facilitate the improvement of ionic conductivity of the SPEs [2,26].

Various salts have been used such as ethylene carbonate (EC), propylene carbonate (PC), carbonate (DEC), and recently silicon dioxide (SiO_2_), TiO_2_, Sb_2_O_3_ and graphene oxide (GO) have similarly been used to improve SPE performance. Previous studies have reported a significant improvement in the amorphous region of the matrix through polymer modification with the incorporation of different salts/plasticizers [17]. The increment in the amorphous region of a matrix is favorable to the motion of the local PVA chain in addition to the water uptake of the electrolytes. This promoted the passage of ions and subsequently improved the conductivities of the electrolytes. An increase in the conductivity of SPE was reported with the incorporation of NH_4_NO_3_ [123].

### 9.2. Blending of Different Polymers

Polymer blending or mixing of two or more polymers is another available method that can be used in producing polymer electrolytes with high electrochemical performance. Polymer blending was reported to be an active way of reducing the crystalline nature of a polymer material. In this method, two or more different polymers are physically mixed via strong stirring to produce new macromolecular material. One of the polymers in the blends is added to provide mechanical strength, while the second is to absorb the active charge carriers in the electrolyte [29]. Blending of two or more polymers to prepare and synthesized SPE does not necessitate the polymerization process and so makes it cost-effective. The physicochemical characteristics of the polymer mixtures rely on the contributing polymers and the solvent. Homogenous polymer blends are produced if two or more different polymers can dissolve successfully and quickly establish thermodynamic equilibrium in a common solvent. However, one common disadvantage of using polymer blends is finding a single solvent that can be able to dissolve two different types of polymers [124].

Blending is a very common method to increase the electrochemical performance of energy storage devices because of the synergetic influence and interactions between two or more polymers. For instance, hydrogen bond formation between polymers can decrease crystallinity and positively affects the ionic conductivity of the electrolyte material. Materials such as copolymers and grafted polymers are self-assembling compounds, which can form ion-conducting chains acting as ion transport channels. A list of promising branched of SPEs blends is reasonably diverse because of the many likely blends of backbones and branching chains, in addition to polymer cores [125].

The conductivity of blend SPE Is due to the movement of charge carriers within the electrodes through the polymer matrix [126]. The chemical coordination of ions with polar groups from the polymer complex results mainly from electrostatic forces owing to the coordinating bond formation [7,8]. There are essential features that influence the polymer–salt (ions) interactions, such as composition, nature of the available functional groups and their distances in the polymer matrix. Furthermore, the type and number of molecular weights of the matrix may influence the coordination between salt and the matrix. The ions mostly cations jump from one spot to another when an electric field is applied.

**Figure 29:**
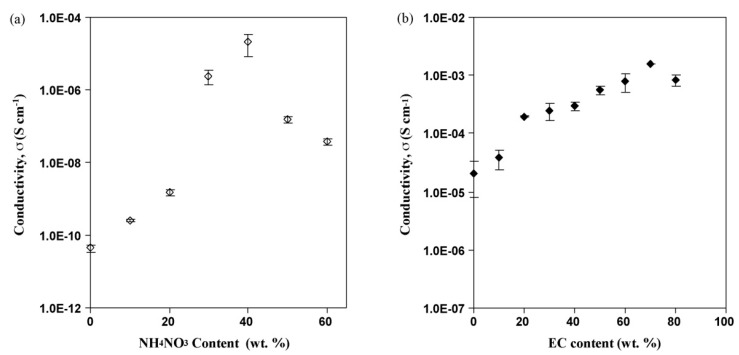
Variation of conductivity of chitosan–PVA on (**a**) NH_4_NO_3_ and (**b**) EC concentrations at room temperature [69].

Many studies have reported the use of polymer blend to prepare SPE based on PVA and other polymers. For instance, Kadir and his co-authors studied the effect of salt content on ionic conductivity of the PVA–chitosan electrolyte [69]. The authors reported that the conductivity of pure chitosan–PVA blend is low at room temperature, as presented in Figure 29. From the figure, the authors observed a significant increase in the conductivity of chitosan–PVA with the incorporation of NH_4_NO_3_ and ethylene carbonate (EC) concentration. The authors attributed the increase in conductivity to the increase in salt concentrations, which can dissociate and provide more ions that contribute to the increase in conductivity. The study reported that, at higher salt concentration in the blend, the rate of ion dissociation is observed to be higher than the rate of association. However, they observed that the ionic conductivity increases progressively with the increase in salt concentration until 40 wt.%. Beyond 40 wt.%, the conductivity decreases, and this may be connected to the distance between dissociated ions becoming too close, such that they are able to recombine and form neutral ion pairs that do not contribute toward conductivity [91]. Thus, the number density of ions in the polymer matrix decreases, which resulted in a decrease in conductivity. Similarly, the mobility of the ions in the polymer blend was found to decrease at a higher salt content, as the increase in salt concentration will increase the viscosity of the solution [92].

Consecutively, a number of ionically conducting polymer blend electrolytes with different combinations of conducting salts and solvents were reported in the literature, as summarized in Table 3. As shown in the Table 3, most of the ionic conductivities of the polymer blend electrolytes are moderately higher compared to most of the electrolytes based on single polymers.

### 9.3. Mixed Salt System

To prevent aggregate and cluster formation which may lead to re-crystallization in SPE, previous studies have developed a new method known as a mixed salt system. Mixing of dual salts in polymer electrolytes enhances the ionic conductivity of SPEs as compared to single salt systems. In one reported investigation, polymer electrolytes based on proton-conducting polymer from PVA, ammonium acetate and 1-butyl-3-methylimidazolium chloride (BmImCl) were reported. The authors established that the electrolyte’s ionic conductivity keeps increasing with the addition of ILs, where the optimum conductivity of 5.74 × 10^−3^ Scm^−1^ was reached at 50 wt% of BmImCl. The authors attributed the increase to the tough plasticizing effect of the ILs [133].

### 9.4. Additives

Several types of additives have been reported to increase the ionic conductivity of an electrolyte, such as plasticizers and fillers, etc.

#### 9.4.1. Plasticizer

A previous study reported that the main drawbacks of using PEs is low conductivity [134]. To solve these problems, many methods were developed to transform the structure of the PEs in order to improve ionic conductivity [135]. One of the most effective ways used is by the addition of plasticizers. Different plasticizers have been used previously such as PEG, EC, PC, DMF, DEC, DMC etc. Plasticization is a vital way to improve the conductivity of PEs. A good plasticizer increases the number of charge carriers (ions) and the interfacial between charge carriers and polar groups of the polymer, which contribute towards the conductivity of the electrolyte [134]. Maximizing the electrolyte conductivity is of significant importance, especially in the PEs materials. The purpose addition of plasticizers to PEs is to increase the amorphous region so that the ionic conductivity is improved [17]. The increase in the amorphous region is favorable to the polymer, which supports ions migration and consequently improves the ionic conductivities of the electrolytes [136]. A previous study has reported an improvement in conductivity with the incorporation of plasticizers, NH_4_NO_3_ [117].

Furthermore, the degree of amorphosity that controls ionic conduction in the PEs can be enhanced greatly with the addition of a plasticizer. For instance, the addition of EC into the PEs was reported to effectively increase ionic conductivity in different types of PEs [123]. It was also proved that the conductivity of PEs improved with the addition of EC where maximum conductivity of 2.0 × 10^−4^ mScm^−1^ was attained. This is due to the high dielectric constant of EC, which enhance polymer segmental motion and as a result increase the conductivity [17].

#### 9.4.2. Filler

Filler is an additive used to increase the mechanical properties of SPEs. The addition of fillers into the PEs leads to the formation of composites [137,138]. A previous study has found that ionic conductivity of nano–sized Al_2_O_3_ incorporated into the PEs was higher than that of micrometer–sized Al_2_O_3_ by almost an order of magnitude. The study further confirmed that the small particle size of fillers improved the electrochemical performance of the PEs by increasing the homogeneity of the samples [83]. Likewise, SiO_2_ is known to be one of the important fillers added to PEs in order to improve electrochemical performance. SiO_2_ is a popular matrix material owing to its mechanical and thermal stability. The effect of adding SiO_2_ nanoparticles into the electrolytes has been investigated, and it was found that the addition of 10 wt% SiO_2_ nanoparticles results in about a one-hundred-times-higher conductivity [77]. The addition of silica into the solid electrolyte was found to reduce Tg and the melting temperature ™ of the composite PEs, which leads to an increase in conductivities [139]. It was reported that the incorporation of silica powders into PEs has two benefits [135]. The first is to improve the conductivity. The mechanism is that the presence of SiO_2_ will inhibit the process of polymer crystallization so that the amorphous region is conserved. Another benefit is that with the addition of SiO_2_, the stability at the electrode–electrolyte interface can be improved.

Similarly, thermal analysis of the electrolytes containing silica indicates an increase in the extent of the interfacial regions. A major improvement in conductivity from 5 × 10^−5^ to 5 × 10^−4^ mS/cm was reported with the incorporation of SiO_2_ particles into PEs. They found that SiO_2_ nanoparticles decrease the ordered structure of the PEs and provide fast movement of ions throughout the conductive pathways. Similarly, another study compared the ionic conductivity of pure PVA with that of SiO_2_ particles, and they reported that the PVA with SiO_2_ particles further increase the ionic conductivity [134]. Fillers such as Al_2_O_3_, SiO_2_, and ZnO were reported to show many advantages in the PEs:

(a) Increase physical properties of polymer matrix;
(b) Reduce the crystallinity and increase the amorphous degree of the PEs;
(c) Decrease glass transition temperature (*T_g_*) of polymer membrane;
(d) Increase the long-term electrochemical stability and electrochemical devices;
(e) Increase the morphological properties of PEs;
(f) Improve the thermal stability of polymer matrix etc. [140].

## 10. Application of PVA-Based Polymer Electrolytes

In this section, the application of PVA-based SPEs in major energy storage devices, such as supercapacitor, LIB, and proton-conducting batteries were extensively reviewed and discussed.

### 10.1. Application of PVA-Based Polymer Electrolytes in Supercapacitor

A recent study has shown that SPEs mostly from PVA are a technique used to improve the physical and mechanical properties of polymer-based electrolytes [140,141,142,143,144]. Many authors reported the use of PVA as host polymer electrolyte for application in SCs. For instance, Kanbara et al. [145] and Lim et al. [90] have tested PVA as a solid polymer electrolyte in EDLC. Both authors established that PVA is well-suited as an electrolyte in EDLC applications with a specific capacitance of 2.5 Fg^−1^. It is also shown that the addition of TiO_2_ particles can enhance the performance in these applications, reaching an electrochemical stability window between −2.3 V and 2.3 V in a cell with carbon electrodes up to 1000 cycles.

**Figure 30:**
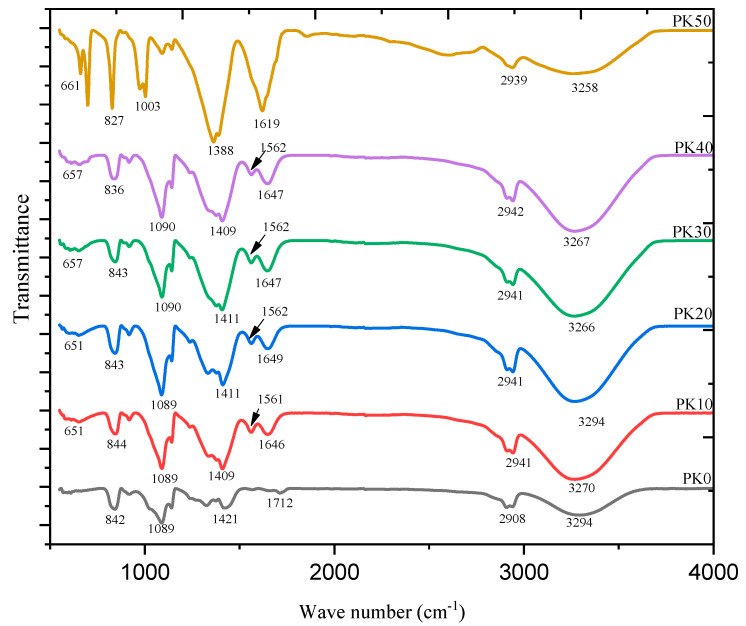
FTIR spectra of pure PVA (PK0), PK10, PK20, PK30, PK40 and PK50 polymer electrolytes [66].

Similarly, Bashir and co-authors reported polymer electrolytes based on PVA in the presence of K_2_CO_3_ salt [66]. The electrolyte was prepared using solution casting technique by incorporating different wt.% (0–50 wt.%) of the conducting salt into the polymer host. The authors confirmed a complex formation between the polymer and the salt, as confirmed by FTIR analysis. As the previous studies reported the addition of the conducting salt into the polymer host, the cation of the metal from the salt would coordinate with the polar groups in the host polymer matrix, which resulted in the complexation [88]. This type of interaction will influence the local structure of the polymer backbone, and certain infrared active modes of vibration will be affected (Figure 30). FTIR spectroscopy is used to analyze the interactions and complexations between atoms or ions in PVA-K_2_CO_3_ electrolyte composites. These interactions can induce changes in the vibrational modes of the polymer electrolyte. Consequently, the changes in the peak intensities and shifting are important in order to establish the complex formation between the host polymer and K_2_CO_3_ [146]. The increased intensities of the peak and shifting in the spectra of the electrolytes confirmed successful formation of PVA-K_2_CO_3_ composites [66].

**Figure 31:**
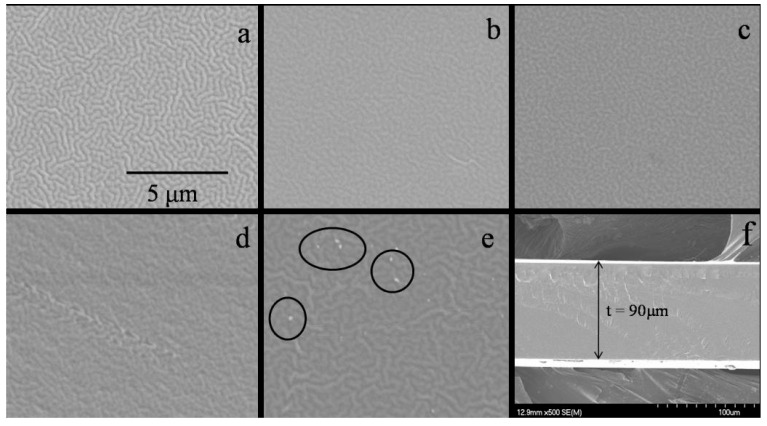
Morphological images of different electrolytes: (**a**) pure PVA, (**b**) P-40, (**c**) P-40-5, (**d**) P-40-10, (**e**) P-40-15 and (**f**) cross-section of P-40-10 [88].

High-performing PVA-based Li-Ion-conducting SPEs films for EDLC applications was reported by Wang and co-authors [88]. The electrolyte was prepared by adding lithium bis (trifluoromethane) sulfonamide (LiTFSI) and 1-ethyl-3 methyl imidazolium bis (trifluoro methyl sulfonyl) imide (EMITFSI) into the PVA matrix by means of the solution casting process. The electrolyte was characterized for their structural and electrochemical performance. The morphologies of the prepared electrolyte are denoted in Figure 31. The authors observed that the electrolyte exhibited a homogeneous and compact wrinkled texture due to the disordered structure of the polymer matrix with the addition of the salts, which confirmed the formation of composites. Similarly, it was found that the PVA-salt electrolyte is capable of coordinating and transporting Li^+^ ions, and it has a relatively wide electrochemical stability window, which is suitable for EDLC applications. The electrolyte was applied in EDLCs and was found to have a specific capacitance of 101 F g^−1^ and an energy density of 10.3 W h kg^−1^, implying that the specific capacitance and energy density retentions are as high as 94.4% and 98.1%, respectively. This demonstrates that PVA-LiTFSI + EMITFSI electrolyte is a favorable electrolyte candidate for electronic device applications.

**Figure 32:**
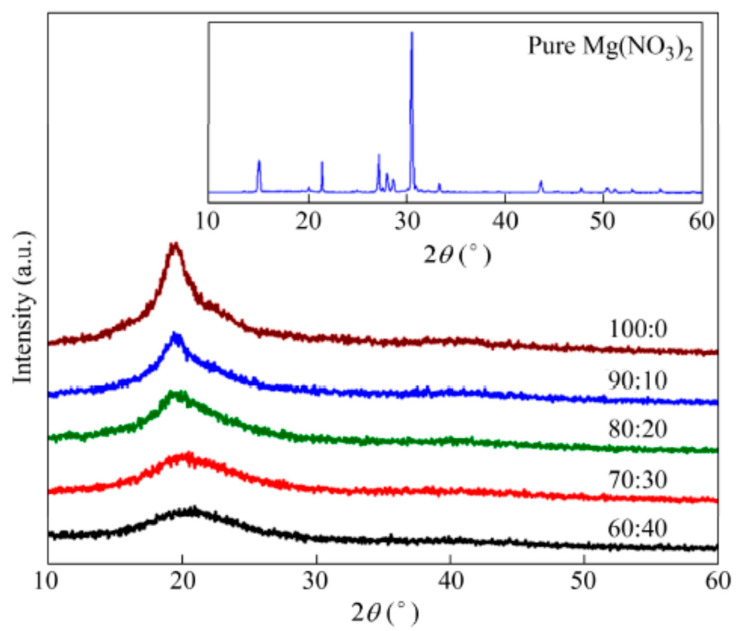
X-ray diffraction patterns of pure Mg(NO_3_)_2_ and PVA:Mg(NO_3_)_2_ complexes [39].

In another study, SPEs containing PVA and magnesium nitrate (Mg(NO_3_)_2_)-conducting salt for application in EDLC were prepared using a solution casting technique and were reported previously [39]. The electrolyte was characterized using XRD, FTIR, DSC and AC impedance spectroscopic analysis, where the complex formation between PVA and Mg salt was confirmed by FTIR and XRD studies. Figure 32 represents the XRD patterns of the prepared electrolytes showing pure PVA, Mg(NO_3_)_2_ and different compositions of PVA-Mg(NO_3_)_2_ polymer electrolytes. The XRD pattern of the PVA electrolyte displays a broad peak between 17° and 22°, which is associated with the amorphous domain of the PVA polymer matrix. The authors found that the PVA matrix combined with Mg(NO_3_)_2_ has a larger domain of amorphous phase, and consequently, the ionic conductivity of the electrolyte can be significantly enhanced. No peaks pertaining to magnesium nitrate salt appeared in XRD patterns of the complexes, which indicated the complete dissolution of magnesium salt in the polymer matrices. The XRD studies confirmed the fact that there exists a definite complex coordination between PVA and magnesium nitrate salt.

**Figure 33:**
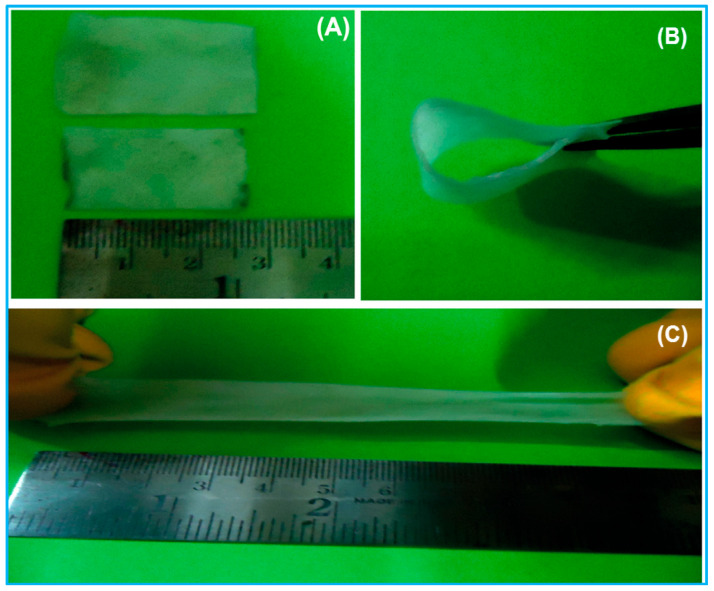
Digital photographs of (**A**) PVA electrolyte, (**B**) flexibility of PVA electrolyte by bending in circular arc and (**C**) PVA electrolyte stretched to check its elastic ability [70].

Chodankar and co-authors developed an ionically conducting polymer electrolyte based on PVA–LiClO_4_ for high-performance flexible supercapacitors. The developed electrolyte was found to demonstrate suitable ionic conductivity comparable with other electrolytes, better compatibility with an active electrode and excellent mechanical feasibility. The electrolyte showed a better operating potential window, high specific capacitance, and high energy density with extended cycling stability up to 2500 CV cycles. Furthermore, the developed electrolyte exhibits enhanced self-discharge time and consistency period. As displayed in Figure 33, the mechanical flexibility of PVA electrolytes was studied by folding and stretching the electrolyte ribbon. Figure 33A displays the digital photograph of the PVA electrolyte ribbon. To check the mechanical flexibility of the sample, the PVA electrolyte is bent at an angle of 180^o^ without any fracturing, as can be seen in Figure 33B. Moreover, the PVA electrolyte is elastically stretched to about three times its original length (Figure 33C). Moreover, it was observed that the electrolyte could recover its original dimension when the externally applied force is removed, indicating the good mechanical features of PVA electrolytes. The excellent mechanical and elastic properties of PVA electrolytes are beneficial to develop a high-performance flexible supercapacitor [70].

**Figure 34:**
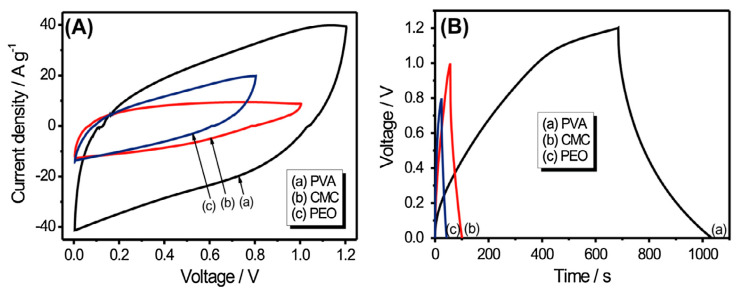
(**A**) The CV curves of fabricated supercapacitor with the developed electrolytes based on PVA, CMC and PEO at a scan rate of 100 mV s^−1^, (**B**) Galvanostatic charge–discharge curves at a current density of 2 A g^−1^ [70].

Cyclic voltammetry (CV) curves using the developed electrolyte at a scan rate of 100 mV s^−1^ within an optimized operating potential window is presented in Figure 34A. The supercapacitor device with PVA electrolyte exhibits a good operating potential window. Furthermore, the supercapacitor device with PVA electrolyte demonstrates a higher area under the CV curves with symmetry along the cathodic and anodic directions signifying good supercapacitive features. Similarly, Figure 34B further reveals the charge–discharge curves of the supercapacitor using PVA-based electrolytes at a constant current density of 2 A g^−1^. The author established that the PVA electrolyte-based supercapacitor shows good performance such as much large discharge time and nearly symmetric behavior of charge–discharge curve, as compared to the supercapacitor device with other electrolytes. The better electrochemical performance of PVA electrolyte-based supercapacitor is due to the higher ionic conductivity, better compatibility and excellent mechanical integrity as compared to the other electrolytes [70].

### 10.2. Application of PVA-Based Polymer Electrolytes in LIB

Lithium-ion batteries are common energy storage devices for electric vehicles, cell phones, laptops, and other portable devices due to their high energy densities, high output voltages, and long cycle lives [147,148]. However, LIBs have some serious safety issues that include electrolyte leakage, unwanted chemical reactions, and explosion due to the formation of lithium dendrite, especially when using liquid electrolytes. To address these issues, SPEs was reported to be safe and suitable electrolytes to use, and this is due to their high energy densities and absence of electrolyte leakage, which remove the flammability hazards [149]. A number of polymers have emerged as excellent candidates for the preparation of SPEs for LIB application; among them, PVA was reported to be one of the best polymers used as a host polymer for the preparation of SPEs for LIB application, due to its good ability to dissolve lithium salts and its good interfacial contact with electrodes [111].

Muthiah and co-authors developed proton-conducting polymer electrolytes based on PVdF-PVA with NH_4_NO_3_ using a solution casting technique and the electrolyte was applied for LiB. This is because it was found that PVA was reported to be a polymer with a carbon chain backbone attached to hydroxyl groups. The optimized PVdF-PVA polymer blend ratio was doped with different concentrations of NH_4_NO_3_. The authors reported an increase in the amorphous nature of the PEs using XRD analysis and optical microscopic studies, while the complex formation between polymers and the salt was confirmed by FTIR analysis. The optical images based on optical microscopic studies of the electrolytes confirmed the complex formation between the polymers and the salt [127].

**Figure 35:**
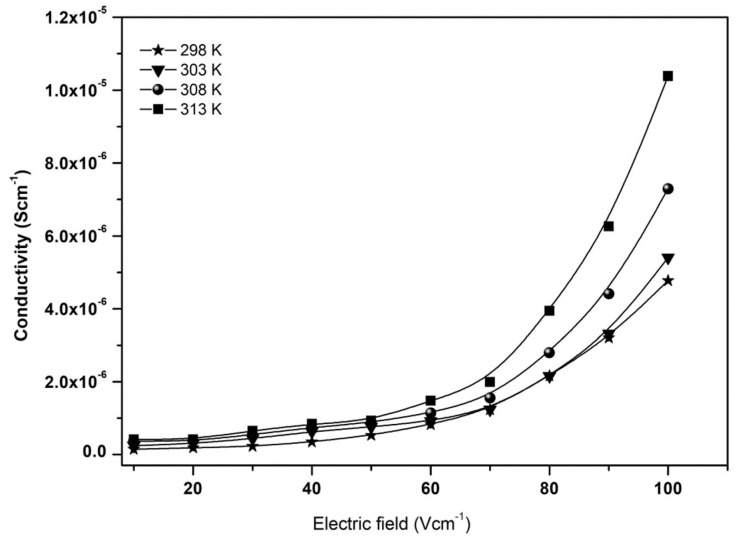
Electric field vs. conductivity of PVA-based electrolyte at different temperatures [99].

In another study, nanocomposite SPEs based on PVA with different combinations of nano ferroelectric fillers for application in LIB were prepared using the solution casting technique, as reported by Sunitha and co-authors [99]. The authors found that the addition of nanofillers to PVA increases the DC conductivity of the SPEs. Similarly, the structural change induced in the electrolyte due to the addition of nanofillers was investigated using XRD techniques, and it was found that the amorphosity of the sample increased as the combination of nanofillers was added to the polymer matrix. The effect of temperature on conductivity was studied in the range of 298–323 K while changing the electric fields from 0 to 100 Vcm−1. It was established that the ionic conductivity of the electrolyte increased non-linearly with the electric field, as depicted in Figure 35.

Similarly, composite polymer electrolytes (CPEs) based on PVA/PAN for application in LIB were reported previously. The authors used a solution casting technique and CPEs based on poly(vinyl alcohol)/polyacrylonitrile were prepared where the electrolyte’s electrochemical stability, ionic transport properties, and interfacial behavior against lithium electrodes were investigated. The study found that the electrochemical stability window of the electrolytes based on PVA as well as Li^+^ -ion transference number significantly improved from 0.16 to 0.507. Likewise, the lithium plating/stripping curve revealed excellent interfacial stability between the electrolyte and the lithium metal electrode. Subsequently, the assembled LIB was found to exhibit excellent cycling and rate performance at room temperature where it delivered a discharge capacity of 159.6 mA h g^−1^ and it continued at 156.9 mA h g^−1^ after 30 cycles, showing capacity retention of 98.3%. Therefore, the authors confirmed that the prepared electrolyte based on PVA can be used as a suitable electrolyte for application in LIBs [111].

### 10.3. Application of PVA-Based Polymer Electrolytes in Proton-Conducting Batteries

**Figure 36:**
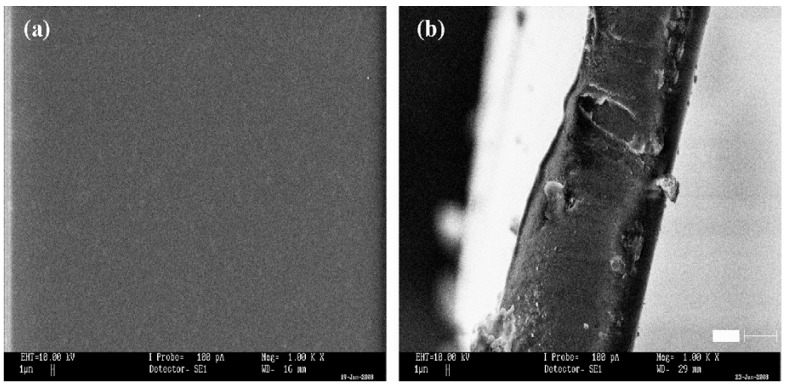
SEM images of pure chitosan–PVA films at (**a**) surface and (**b**) cross-section [69].

Transport studies of PVA–chitosan polymer electrolyte for application in proton batteries was reported by Kadir and co-authors [69]. The solution casting technique was used and prepared the electrolytes system. The authors blended 36 wt.% of PVA with 24 wt.% chitosan and doped with 40 wt.% NH_4_NO_3_ as a source of ions. Based on the SEM surface and cross-section micrographs result of chitosan–PVA blended electrolyte (shown in Figure 36), the film displays a smooth and homogeneous surface indicating that both polymers are miscible with each other.

In another study, it was reported that for PVA modified by chitosan, the chitosan adhered to the inner surface of the interconnected pore structure of PVA. Thus, it might be concluded that chitosan will be inserted in the pores of PVA. The micrograph of the cross-section is proof of the miscibility of chitosan and PVA. The highest conductivity obtained in the prepared polymer blend electrolyte was 1.60 × 10^−3^ Scm^−1^. Subsequently, the authors applied the Rice and Roth model, whereby the conductivity enhancement was analyzed, and the highest conducting sample was used, and several batteries were fabricated with configuration Zn//MnO_2_. The open circuit potential (OCP) of the fabricated batteries was between 1.6 and 1.7 V [69].

**Figure 37:**
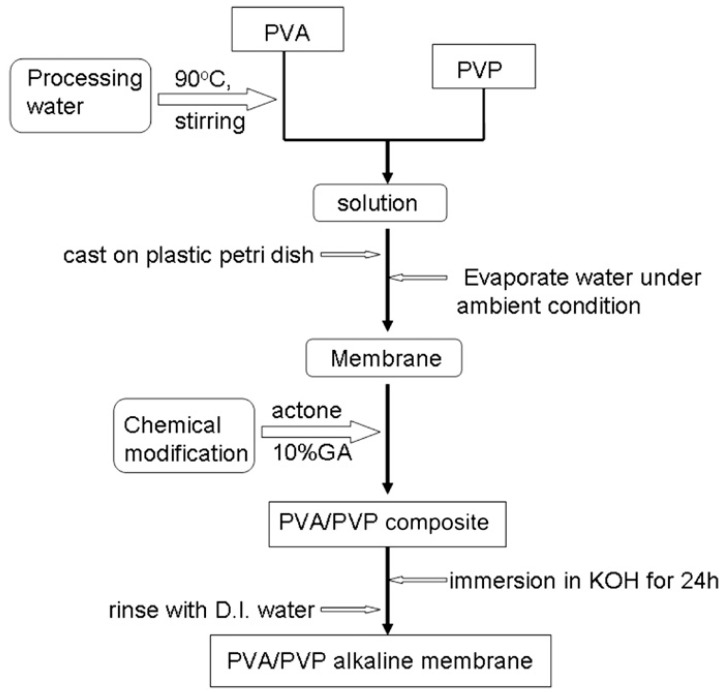
The schematic diagram for the preparation of PVA/PVP by blending and chemical cross-linking [128].

A novel alkaline solid polymer electrolyte membrane that can conduct anions (OH^−^) was developed based on PVA/PVP blending and chemical cross-linking with the incorporation of KOH as the source of ions where PVP serves as both a plasticizer and stabilizer. Figure 37 schematically demonstrates the preparation of chemically cross-linked PVA/PVP blended electrolytes. The physicochemical properties of the samples were investigated by FTIR, TG, and SEM analyses. The membrane showed flawless alkaline strength without losing its integrity, even upon exposure to high concentrations of KOH solution and at a higher temperature. Morphological analysis by SEM revealed a highly ordered micro-void structure that was homogeneously distributed on the surface of the membrane, which conveyed the membrane with good electrolyte (KOH) retention ability. FTIR spectra confirmed that the formation of a good complex might be ascribed to the presence of excess free KOH in the polymer matrix in addition to KOH bound to the polymers. The electrolyte was applied in proton-conducting batteries application where its electrochemical performance of the electrolytes was found to be significantly dependent on the concentration of KOH and the PVP content. Virtually continuous, highly stable ionic conductivity while maintaining mechanical integrity was retained at room temperature for more than one month [128].

## 11. Challenges and Future Directions

It has been shown in the previous sections that electrolytes, especially those from PVA, play an important role in the supercapacitor’s performance. Apart from their cost, their associated technical parameters such as ionic conductivity, potential window and the mechanical integrity of PVA-based SPEs are important and essential considerations to fabricate high-energy-density, lightweight, flexible, safe, leak-free and durable next-generation solid-state supercapacitors. Several research groups are working hard toward controlling the advanced aspects of PVA-based SPEs such as optimizing the properties and improving the electrochemical performances. PVA-based SPEs have been identified as key components in supercapacitor performance. The values of electrolyte stable potential window, ionic conductivity, chemical and thermal stabilities, as well as operating temperature range, have significant effects on both SPE performance and practical applications.

Although great progress has been made in the field of PVA-based SPEs, there are still a number of major challenges which hinder the development of the technology and its commercial applications [17,150]. Such challenges are summarized as follows: low ionic conductivity and insufficient potential window values and their effect on energy and power density, and un-optimized salt concentration having maximum conductivity in a PVA salt-based electrolyte.

To overcome these challenges, several future research directions are suggested as follows: improving PVA-based SPE’s ionic conductivity and potential window values in order to increase supercapacitors’ energy and power density through the use of appropriate modifiers (blend with other polymers) and additives (plasticizers and fillers) to further increase the supercapacitor electrochemical performance. Critical research on the optimization of the conducting salt based on SPEs is also required. Further fundamental understanding of SPEs through both theoretical and experimental investigations, and the development of standard methods to evaluate the performance of SPEs, is suggested.

## 12. Conclusions

To identify the research direction and development of PVA-based SPEs for supercapacitor application, this paper provided a critical overview of the recent trends and development in PVA-based SPEs. The impact of PVA-based SPE’s performance on supercapacitor’s performances was explained. Various types of solid electrolytes and their application particularly in supercapacitors and a few other energy storage devices were discussed, and their effects on supercapacitor performance were reviewed. Polymer–salt interactions in PVA-based SPEs on ionic conduction mechanism were highlighted, where important parameters that govern ionic conductivity and charge carriers were extensively discussed. Thermodynamic ion transport models for electrolytes were explained, and the ways to enhance the ionic conductivity of PVA-based SPEs were explored and well explained. The fundamental relationship between impedance plots and linear sweep voltammetry, which are critical for electrochemical characterization, was also analyzed and discussed in detail. The challenges facing PVA-based SPEs in relation to supercapacitor performance were identified, and possible research solutions and directions are proposed to overcome these challenges in future studies with the purpose of improving the supercapacitor’s maximum performance.

## Figures and Tables

**Table 1:** Ionic conductivity of some polymer electrolytes based on PVA and their corresponding conducting salts.

S/N	Polymer	Salts	Ionic Conductivity (σ)	References
1	PVA	NH_4_I	2.50 × 10^−3^ Scm^−1^	[68]
2	PVA	NH_4_Br	5.70 ×10^−4^ S cm^−1^	[68]
3	PVA	NH_4_Cl	1.00 × 10^−5^ S cm^−1^	[68]
4	PVA	Mg(NO_3_)_2_	7.36 × 10^−7^ S/cm	[39]
5	PVA	NH_4_NO_3_	2.07 × 10^−5^ Scm^−1^	[69]
6	PVA	CH_3_COONH_4_	5.62 × 10^−6^ Scm^−1^	[94]
7	PVA	Cu(NO_3_)_2_	1.60 × 10^−5^ Scm^−1^	[95]
8	PVA	AgNO_3_	7.56 × 10^−7^ Scm^−1^	[96]
9	PVA	K_2_CO_3_	4.53 × 10^−3^ Scm^−1^	[66]
10	PVA	H_3_PO_4_	2.50 × 10^−3^ Scm^−1^	[97]
11	PVA	LiAsF-TiO_2_	5.10 × 10^−4^ Scm^−1^	[98]
12	PVA	KI-I_2_	8.41 × 10^−3^ Scm^−1^	[51]
13	PVA	NH_4_NO_3_	1.60 × 10^−3^ Scm^−1^	[67]
14	PVA	TiO_3_-SrTiO_3_-Al_2_O	5.20 × 10^−5^ Scm^−1^	[99]
15	PVA	NaI	2.41 × 10^−4^ Scm^−1^	[100]
16	PVA	LiClO_4_	4.80 × 10^−3^ Scm^−1^	[70]
17	PVA	LiTFSI-EMITFSI	5.30 × 10^−7^ Scm^−1^	[88]
18	PVA	LiClO_4_-TiO_2_	1.30 × 10^−4^ Scm^−1^	[90]
19	PVA	BC	6.63 × 10^−2^ Scm^−1^	[54]
20	PVA	NaClO_4_-LiClO_4_	1.57 × 10^−3^ Scm^−1^	[101]
20	PVA	H_3_PO_4_	8.06 × 10^−5^ Scm^−1^	[102]
22	PVA	K_2_CO_3_-SiO_2_	7.86 × 10^−3^ Scm^−1^	[75]
23	PVA	Ce(III)-NH_4_SCN	2.07 × 10^−3^ Scm^−1^	[103]
24	PVA	SiO_2_	2.0 × 10^−2^ Scm^−1^	[104]

**Table 2:** Potential window of some polymer electrolytes based on PVA and their corresponding conducting salts.

Polymers	Salts	Potential Window (V)	References
PVA	LiCLO_4_-TiO_2_	4.60	[90]
PVA	K_2_CO_3_	2.70	[66]
PVA	NH_4_NO_3_	3.30	[69]
PVA/PAN	LATP	5.10	[111]
PVA	LiTFSI-EMITFSI	5.00	[88]
PVA	Ce(III)-NH_4_SCN	2.11	[103]
PVA	K_2_CO_3_-SiO_2_	3.35	[75]
PVA–chitosan	NH_4_NO_3_	1.70	[112]

**Table 3:** Summary of the ionic conductivities with different conducting salts and solvents based on polymer blend electrolytes.

Polymers	Salt	Solvents	Conductivity (S/cm)	References
PVdF-PVA	NH_4_NO_3_	DMF	2.91 × 10^−4^	[127]
PVA/PVP	KOH	Distilled water	0.53	[128]
CS/PVA	NH_4_NO_3_	Acetic acid	1.60 × 10^−3^	[69]
PVA-MC	NH_4_SCN	Distilled water	1.45 × 10^−4^	[129]
PVdF-PVA	NH_4_SCN	DMF	1.09 × 10^−3^	[130]
PVA–PMMA	LiBF_4_	DMF	1.28 × 10^−3^	[131]
PVA-PEO-PVdF	NaOH	H_2_SO_4_	0.18 × 10^−5^	[72]
PVA-MC	NH_4_NO_3_	Distilled water	7.39 × 10^−8^	[114]
PVA-MC	AuNPs	Distilled water	3.08 × 10^−8^	[132]

## Data Availability

Not applicable.
